# Structural insights into the Clp protein degradation machinery

**DOI:** 10.1128/mbio.00031-24

**Published:** 2024-03-19

**Authors:** Xiaolong Xu, Yanhui Wang, Wei Huang, Danyang Li, Zixin Deng, Feng Long

**Affiliations:** 1 Department of Neurosurgery, Zhongnan Hospital of Wuhan University, School of Pharmaceutical Sciences, Wuhan University, Wuhan, China; 2 Ministry of Education Key Laboratory of Combinatorial Biosynthesis and Drug Discovery, School of Pharmaceutical Sciences, Wuhan University, Wuhan, China; 3 Cryo-EM Center and the Core Facility of Wuhan University, Wuhan, China; Case Western Reserve University School of Medicine, Ohio, USA

**Keywords:** ClpP protease, AAA+ ATPase, protein degradation, ADEP1, substrate translocation mechanism

## Abstract

The Clp protease system is important for maintaining proteostasis in bacteria. It consists of ClpP serine proteases and an AAA+ Clp-ATPase such as ClpC1. The hexameric ATPase ClpC1 utilizes the energy of ATP binding and hydrolysis to engage, unfold, and translocate substrates into the proteolytic chamber of homo- or hetero-tetradecameric ClpP for degradation. The assembly between the hetero-tetradecameric ClpP1P2 chamber and the Clp-ATPases containing tandem ATPase domains from the same species has not been studied in depth. Here, we present cryo-EM structures of the substrate-bound ClpC1:shClpP1P2 from *Streptomyces hawaiiensis*, and shClpP1P2 in complex with ADEP1, a natural compound produced by *S. hawaiiensis* and known to cause over-activation and dysregulation of the ClpP proteolytic core chamber. Our structures provide detailed information on the shClpP1-shClpP2, shClpP2-ClpC1, and ADEP1-shClpP1/P2 interactions, reveal conformational transition of ClpC1 during the substrate translocation, and capture a rotational ATP hydrolysis mechanism likely dominated by the D1 ATPase activity of chaperones.

**IMPORTANCE:**

The Clp-dependent proteolysis plays an important role in bacterial homeostasis and pathogenesis. The ClpP protease system is an effective drug target for antibacterial therapy. *Streptomyces hawaiiensis* can produce a class of potent acyldepsipeptide antibiotics such as ADEP1, which could affect the ClpP protease activity. Although *S. hawaiiensis* hosts one of the most intricate ClpP systems in nature, very little was known about its Clp protease mechanism and the impact of ADEP molecules on ClpP. The significance of our research is in dissecting the functional mechanism of the assembled Clp degradation machinery, as well as the interaction between ADEP1 and the ClpP proteolytic chamber, by solving high-resolution structures of the substrate-bound Clp system in *S. hawaiiensis*. The findings shed light on our understanding of the Clp-dependent proteolysis in bacteria, which will enhance the development of antimicrobial drugs targeting the Clp protease system, and help fighting against bacterial multidrug resistance.

## INTRODUCTION

The regulated proteolysis is important for maintaining cellular proteostasis by controlling protein turnover and eliminating misfolded and dysfunctional proteins, in response to cellular events, environmental changes, and exogenous invasion (1). Loss of proteostasis is associated with proteotoxic stress, cell death, and many diseases including neurodegenerative disorders such as Alzheimer’s and Parkinson’s disease (2). Proteases belonging to the ATPases associated with diverse cellular activities (AAA+) superfamily are found playing a key role in proteolysis in all life kingdoms (3, 4). The most studied AAA+ protease systems include the eukaryotic 26S proteasome and a number of bacterial proteases such as the caseinolytic protease (Clp), Deg, Lon, and FtsH protease machineries (5–8). About 80% of the cellular proteolysis in bacteria relies on the proteases Clp and Lon (9). The bacterial Clp degradation machinery is generally composed of a main tetradecameric peptidase chamber, formed by stacking of two heptameric ClpP rings, and single- or double-coupled hexameric caps, consisting of AAA+ unfoldase chaperones also known as Hsp100 class of ATPases such as ClpX and ClpC (10).

**Fig 1:**
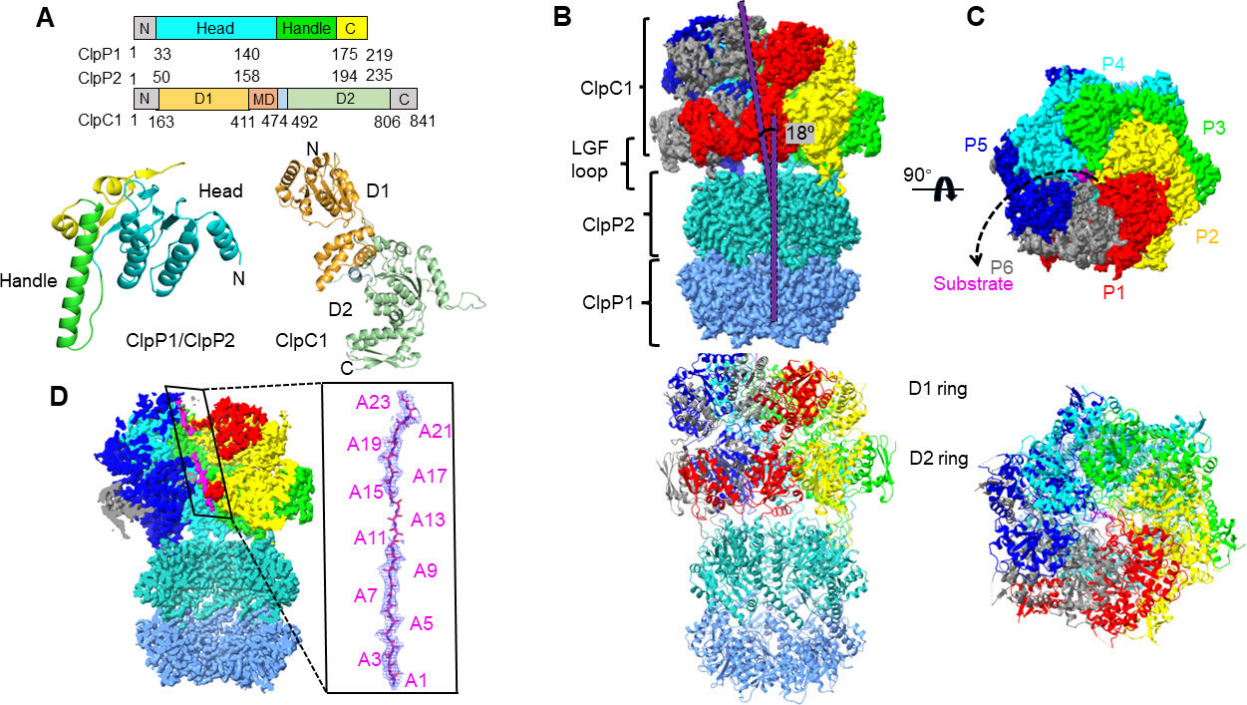
Overview of the ClpC1:shClpP1P2 complex. (A) Linear representation of domain organization of shClpP1P2 and ClpC1. (**B–C)** Overall architecture of the substrate-bound ClpC1:shClpP1P2 complex. The side view (left) and top view (right) are shown. The ClpC1 protomers are individually colored. (**D)** Cross section of ClpC1:shClpP1P2 with a polypeptide substrate engaged by ClpC1 in the central channel.

ClpP is a serine protease that is highly conserved among different species. Many bacterial species, such as *Escherichia coli* and *Staphylococcus aureus*, harbor a single *clpP* gene (11). However, a few bacteria including *Pseudomonas aeruginosa* and *Streptomyces* sp. encode two or more ClpP isoforms, namely ClpP1, ClpP2, and so on (12, 13). The ClpP protomer consists of three discrete domains, including the N-terminal loop, the head domain, and the handle domain (Fig. 1A). ClpP forms the heptameric ring stabilized by extensive interaction between the head domains. Two heptameric ClpP rings are then interlocked between the handle domains to form the functionally active homogeneous (ClpP-ClpP) or heterogeneous (ClpP1-ClpP2) tetradecamers (14). The ClpP tetradecamer chamber might be contractable for function, and three different conformations representing extended active state, compressed inactive state, or compact inactive state had been observed in various Clp systems (15–19). Substrate access to the proteolytic chamber is tightly controlled by the axial pores of ClpP, which restricts the size of the protein substrates degraded by ClpP and only allows small unfolded peptides to enter the secluded proteolytic chamber for degradation (20).

The engagement, unfolding, and translocation of substrates are usually driven by the ATP hydrolysis of the hexameric AAA+ ATPases. There are two groups of the Clp/Hsp100 family ATPases, including the class 1 ATPases such as ClpA, ClpB, and ClpC that contain two ATPase domains (D1 and D2) and the class 2 ATPases such as ClpX with only one ATPase domain (21). Docking of these Clp ATPases onto the ClpP chamber involves the interaction between a highly conserved Ile/Leu-Gly-Phe motif (I/LGF loops) of the Clp ATPase and an apical hydrophobic pocket on the ClpP surface. Previous studies of Hsp104 in *Saccharomyces cerevisiae* have demonstrated a ratchet-like mechanism in various substrate-binding states (22). The spiral arrangement of the hexameric Clp ATPase favors a rotational ATP hydrolysis cycle, facilitating substrate release by the protomer at low positions and subsequent rebinding along the substrate when the protomer reaches the top position. Recently, similar spiral structures and substrate contact arrays have been discovered in several other AAA+ machineries, including ClpB (23) and ClpA (24) from *E. coli*. These findings provide further support for a rotational translocation mechanism that is prevalent among AAA+ machineries.

The ClpP-ATPase degradation complexes have been established as important drug targets for the treatment of infection and cancer (25). Several natural product antibiotics, including ecumicin, cyclomarin, lassomycin, and rufomycin, were found to prohibit the cell growth probably by targeting the ClpP-ATPase complex and accelerating protein degradation (26, 27). Another remarkable natural product affecting ClpP is the cyclic acyldepsipeptide (ADEP) antibiotics produced by *Streptomyces hawaiiensis*. ADEP could interrupt the ClpP-ATPase interaction by binding to the peripheral hydrophobic clefts on the ClpP surface and may induce the consistent opening of the ClpP axial pores, leading to unregulated protein degradation and cell death (28–30). A recent study on the *Streptomyces* ClpP system showed that binding of ADEP in ClpP1 could accelerate the ATPase-dependent degradation of the natural substrates (13). However, the detailed mechanism of ADEP1 on the ClpP system remains obscure.

In the past few years, the structures of the degradation ClpP chamber and the individual ATPase chaperones from different species have been studied. Degradation mechanism of the intricate Clp protease system remains elusive due to lack of detailed structural information of the assembled complexes. Up to date, only a few ternary structures of the ATPase-ClpP degradation machinery are solved, including ClpX:ClpP from *Neisseria meningitidis* (19), ClpX:ClpP1P2 from *Listeria monocytogenes* (18), and ClpA:ClpP (24) and ClpS:ClpA:ClpP (31) from *E. coli*. The *Streptomycetes* strains host most redundant ClpP systems among microorganisms, encoding three or five ClpP homologs (30). Here, we determined the first to date cryo-EM structures of the house-keeping Clp protease system (shClpP1P2) and its assembled AAA+ ATPase chaperone ClpC1 from *S. hawaiiensis* NRRL 15010, revealing assembly of the hetero-tetradecameric shClpP1P2 at a near atomic resolution, structural influence of ADEP1 on the shClpP1P2 chamber, and conformational transition of the ClpC1:shClpP1P2 complex during the substrate translocation and ATP hydrolysis by ClpC1. Our findings provide structural insights into the bacterial ClpP-ATPase degradation machinery.

## RESULTS

### Assembly of the ClpC1:shClpP1P2 protease system

To increase success possibility in reconstitution of the shClpP1P2-ClpC1 protease system, we first set out to purify and assemble the binary protein complex of shClpP1P2. However, we encountered difficulties in expressing the full-length shClpP1 and shClpP2 proteins in *E. coli*. No expression of shClpP1 and precipitation of shClpP2 were observed during our expression trials. Removal of the N-terminal flexible regions (first 29 residues removed in shClpP1 and first 49 residues removed in shClpP2) improved solubility of the expressed recombinant proteins (Fig. 1A). Since the truncated proteins yielded poor homogeneity in the assembly of shClpP1P2 (Fig. S1A), single mutations of the conserved catalytic residue (S113A in shClpP1 and S131A in shClpP2) were further introduced to stabilize the stacked ring structure by attenuation of the complex activity (Fig. S2A). The two proteins were separately purified and *in vitro* assembled into the shClpP1P2 complex for cryo-EM analysis (Fig. S1B). The reconstruction of the assembled shClpP1P2 chamber was obtained at a resolution of 2.88 Å with an imposed C7 symmetry (Fig. S3A).

The purified full-length ClpC1 was uncapable of forming a steady hexamer in solution (Fig. S1A). Therefore, the modified ClpC1 with less catalytic activity was sought for due to its promising increase of homogeneity. A triple mutant of ClpC1 was constructed with two Walker-B mutations (E284A/E622A) and one MD domain mutation (F440A) (Fig. S2C). The Walker-B mutations were expected to decrease the catalytic activity of ATP hydrolysis but preserve the capability of phosphate nucleotide binding (32). The MD domain mutation may trap the ATPase in a derepressed state (23). Moreover, the NTD domain (residues 1–156) of ClpC1 involved in adaptor binding was removed because it is usually invisible due to its flexibility. The resulting ClpC1 was used to form a complex with shClpP1P2 in the presence of the supplemented fluorescein-labeled casein substrate (33) and ATP (Fig. S1C). The cryo-EM data analysis revealed the existence of both assembled ClpC1:shClpP1P2 and free unassembled ClpC1 or shClpP1P2 in the sample (Fig. S4A).

The reconstruction of the ClpC1:shClpP1P2 ternary complex was conducted and subjected to several rounds of 3D classification. Two distinct groups of ClpC1:shClpP1P2 were obtained at the resolutions of ~3.0 Å, referred as conformation A and conformation B, respectively (Table 1; Fig. S3B). Local refinements of ClpC1 and shClpP1P2 were performed to improve the resolution and map quality, yielding regional maps of ClpC1 and shClpP1P2 that were consequently combined for atomic model building and real space refinement of the models (Fig. S4A). Comparing the two conformations, the hexameric spiral cap of ClpC1 is anchored differently to the shClpP1P2 chamber. Reconstruction of the free unassembled ClpC1 was suspended due to a severe issue of sample orientation preference. However, the free shClpP1P2 particles resulted in a C7 symmetric reconstruction at an overall resolution of 2.34 Å (Fig. S3C).

**TABLE 1:** Cryo-EM data collection, refinement, and validation statistics

	shClpP1P2 PDB: 8XN4 EMD-38497	ADEP1:shClpP1P2 PDB: 8XOP EMD-38537	ClpC1:shClpP1P2 Conformation A PDB: 8XOO EMD-38536	ClpC1:shClpP1P2 Conformation B PDB: 8XON EMD-38535
Data collection and processing				
Magnification	105,000	105,000	105,000	105,000
Voltage (kV)	300	300	300	300
Electron exposure (e^−^/Å^2^)	50	50	50	50
Defocus range (μm)	−1 to −2	−1 to −2	−1 to −2	−1 to −2
Pixel size (Å)	0.84	0.84	0.84	0.84
Symmetry imposed	C7	C7	C1	C1
Initial particle images (no.)	1,481,298	669,496	1,481,298	1,481,298
Final particle image (no.)	258,973	504,306	117,399	73,531
Map resolution (Å)				
Masked (gold standard)	2.34	2.8	2.94	3.0
Unmasked (gold standard)	2.8	3.2	3.6	3.8
FSC threshold	0.143	0.143	0.143	0.143
Map resolution range (Å)	2.2–2.8	2.5–3.2	2.9–5.1	2.9–5.5
Refinement				
Map sharpening *B* factor (Å^2^)	−91.4	−148.0	−68.3	−64.3
Model composition				
Protein residues	2,506	2,478	6,042	6,068
Ligand	0	ADEP1: 14	ADP: 5 ATP(Mg^2+^): 7(7)	ADP: 4 ATP(Mg^2+^): 8(7)
Validation				
Model-map CC**_mask_** (correlation coefficients)	0.84	0.87	0.87	0.87
R.m.s. deviations				
Bond lengths (Å)	0.003	0.003	0.003	0.003
Bond angles (°)	0.560	0.646	0.590	0.568
MolProbity score	1.39	1.47	1.74	1.73
Clashscore	7.09	8.70	8.63	7.93
Rotamer outliers (%)	0.05	0.00	0.02	0.00
Ramachandran plot				
Favored (%)	98.35	98.24	95.97	95.69
Allowed (%)	1.61	1.76	3.85	4.16
Disallowed (%)	0.04	0.00	0.18	0.15

### Structure of the shClpP1P2 chamber

**Fig 2:**
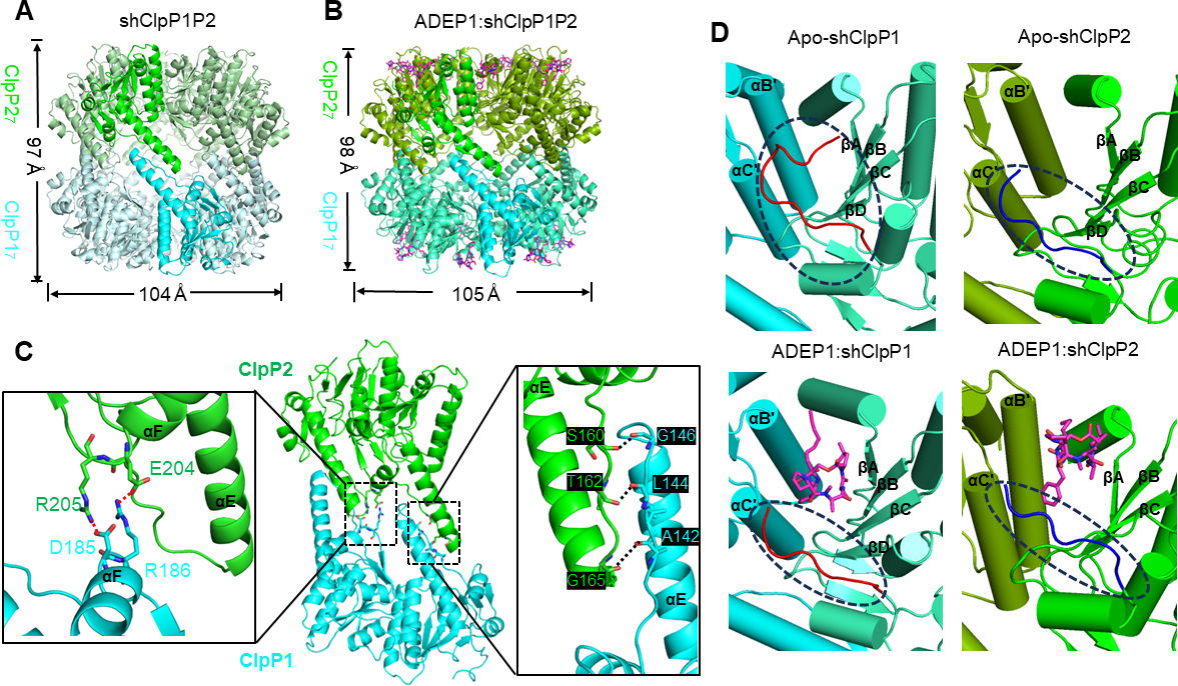
Structures of apo-shClpP1P2 and the ADEP1:shClpP1P2 complex. (A–B) The tetradecameric structures of apo-shClpP1P2 (**A**) and ADEP1:shClpP1P2 (**B**). The ADEP1 molecules are shown as magenta sticks. (**C)** The intermolecular interactions between shClpP1 and shClpP2 stabilize the ring-ring interface of shClpP1P2. The hydrogen bonds and salt bridges are indicated by the dash lines between the interactive residues in the enlarged diagrams. (**D)** Apical hydrophobic pockets formed by two adjacent protomers of shClpP1 (left) or shClpP2 (right). The hydrophobic pockets are covered by the C-terminus of shClpP1 or shClpP2 (enclosed by dashed ovals) in the apo form (upper panel). Upon binding of ADEP1 (in magenta), the C-termini are expelled from the hydrophobic pockets (bottom panel). The key secondary structural elements are labeled.

Since there is almost no difference between the two shClpP1P2 reconstructions that were obtained above from two data sets (RMSD ~0.594 Å), the reconstruction with a better resolution was used for the following structure determination and analysis. The assembled shClpP1P2 chamber is likely in the active extended conformation, consisting of two heptameric stacking rings that are formed by shClpP1 and shClpP2, respectively (Fig. 2A). Numerous hydrogen bonds and salt bridges were detected within the interfaces between the adjacent protomers of shClpP1 and shClpP2, accounting for the inter- and intra-ring stabilization (Table 2; Fig. 2C). The cross-interactive handle domains of shClpP1 and shClpP2 are various in sequences, which are also different from other homo-tetradecamer-forming ClpPs, such as ecClpP and saClpP (Fig. S2B). The specialty of handle regions may determine whether to form homo- or hetero-tetradecamer ClpPs.

**TABLE 2:** Intermolecular interactions in the shClpP1P2 complex

Intermolecular interfaces		Type of interactions
shClpP1-shClpP1′		
Glu134	Arg157	Hydrogen bonds, salt bridges
Arg186	Asp150	
shClpP2-shClpP2′		
Lys118	Arg227	Hydrogen bonds, salt bridges
Asp169	Arg205	
shClpP1-shClpP2 (shClpP2′)		
Pro140	Val147	Hydrogen bonds
Ser141	Arg164	
Ala142	Gly165	
Leu144	Thr162	
Gly146	Ser160	
Ala148	Pro158	
Ser149	Gln157, Glu204	
D185	R205 (shClpP2′)	Hydrogen bonds, salt bridges
R186	E204 (shClpP2′)	Hydrogen bonds, salt bridges

### Structure of shClpP1P2 in complex with ADEP1

The shClpP1P2 structure in complex with ADEP1 was obtained at a resolution of 2.8 Å (Fig. S4B and S5A). A total of 14 ADEP1 molecules were built into the shClpP1P2 structure, with one molecule bound within each apical hydrophobic pocket formed by two adjacent shClpP1 or shClpP2 protomers (Fig. 2B; Fig. S3D). The hydrophobic pocket is located within the cleft formed by a β-sheet (βA, βB, βC) and the C-terminus from one protomer, and two α-helices (αB′, αC′) from the clockwise adjacent protomer (Fig. 2D). The key residues of the ClpP hydrophobic pockets (Y76, Y78, Y98′ in shClpP1 and S94, Y96, Y116′ in shClpP2) are generally conserved among ClpP homologs. Most of the key residues in the hydrophobic pocket of shClpP1/P2 are involved in the interaction with ADEP1 (Fig. S5B). In the apo form of shClpP1P2, the hydrophobic cleft is covered by the C-terminus of shClpP1/P2, which may protect the complex against the hydrophilic environment. Upon the binding of ADEP1, the C-terminus was expelled out of the hydrophobic cleft (Fig. 2D). Compared with apo-shClpP1P2, binding of ADEP1 causes slight dilation of the central pores of both the shClpP1 and shClpP2 rings (Fig. S5D), which is consistent with previous studies of other ClpPs (28). This may facilitate the entry of small unfolded peptides into the proteolytic compartment.

### Architecture of the ClpC1:shClpP1P2 protease system

Local resolution estimation showed that the resolutions of the hexameric ClpC1 are significantly lower than those of the shClpP1P2 chamber in the two ClpC1:shClpP1P2 conformations, indicating structural flexibility and potential dynamic states of ClpC1 (Fig. S3B). In both conformations of ClpC1:shClpP1P2, the six protomers of ClpC1 are arranged stepwise along a central axis, which is tilted ~18° away from the central axis of the shClpP1P2 chamber, forming a shallow spiral dome structure that is hooked onto the apical surface of shClpP1P2 (Fig. 1B). Based on the positions along the spiral axis, the six ClpC1 protomers are counterclockwise labeled from O/P1 (chain O at the lowest position P1) to T/P6 (chain T at the seam position P6 connecting P1 and P5) (Fig. 1C). The chamber structures of shClpP1P2 are nearly identical between two conformations (RMSD ~0.253 Å). The main difference between conformation A and B is the various positional arrangement of the seam protomers in the hexamer ClpC1 structure.

The ClpC1 protomer contains two ATPase domains, D1 and D2, forming the distal and proximal ring-like structures relative to the shClpP2 surface, respectively. The large subdomains of ClpC1 face inside to the central channel, while the small subdomains face outside (Fig. S6B). Although the MD domains are barely visible in the structure, they are mostly extended away from the ClpC1 hexamer shown as the fussy comet tails in the reconstruction (Fig. S6A). The six subunits of ClpC1 are not structurally identical within the hexameric structure. The ClpC1 protomers P2, P3, and P4 are very similar, whereas major displacements of subdomains are observed among the protomers P1, P5, and P6 (Fig. S6C and D). These three seam protomers of ClpC1 are also involved in structural transition from conformation A to B (Video S1). Individual superposition of the six corresponding ClpC1 protomers from two conformations further indicates that the protomers P5, P6, and P1 bear most structural rearrangements of the D1 domains during the conformational transition (Fig. S6E). Thus, it could be inferred that activity status of the D1 ATPases may play an important role in the conformational transition for function of the whole proteolytic Clp machinery.

### Dissection of the ClpC1:shClpP2 interaction

**Fig 3:**
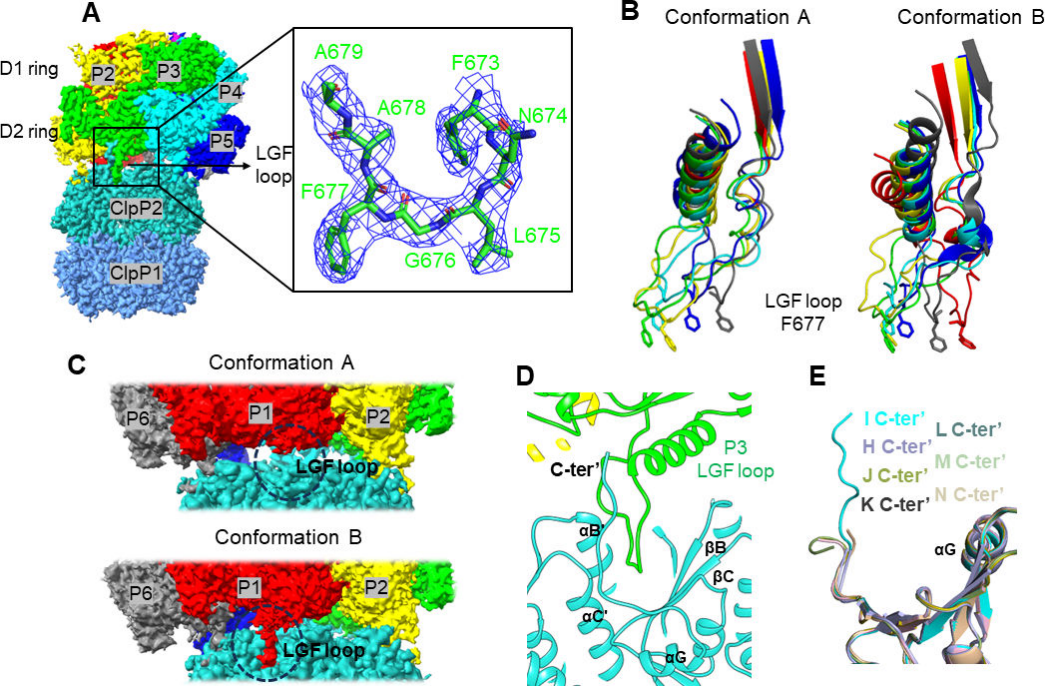
Interaction between the LGF loop of ClpC1 and the hydrophobic pocket of shClpP2 in conformation A and B. (A) The bound ClpC1 LGF loop is shown in the cryo-EM density contoured at 3σ. (**B)** Superimposition of the LGF loops of the ClpC1 protomers in conformation A or B. The aromatic residues Phe677 are shown in sticks for figure clarification. (**C)** The LGF loop of ClpC1 at the P1 position is docked onto the shClpP2 surface in conformation B but undocked in conformation A. (**D)** The LGF loop of ClpC1 at the P3 position is bound within a hydrophobic pocket on the shClpP2 surface and stabilized by the extending C-terminus of shClpP2. (**E)** The C-terminal structures of the seven shClpP2 subunits are superimposed, indicating different conformations of the C-termini of shClpP2.

The attachment of ClpC1 to the shClpP1P2 protease system is achieved by inserting the LGF loop from the D2 domain of ClpC1 into the hydrophobic pocket created by two adjacent shClpP2 protomers (Fig. 3A). Due to the symmetry mismatch between the ClpC1 and shClpP2 subunits, not all seven of the hydrophobic pockets of shClpP2 are occupied by the LGF loops of ClpC1 (Fig. S7A), and the LGF loops of ClpC1 exhibit remarkable plasticity (Fig. 3B). The conserved LGF residues of all the bound ClpC1 loops can be clearly defined in densities (Fig. S7C). Besides hydrophobic interaction, the LGF loop of ClpC1 is stabilized by forming hydrogen bonds with the shClpP2 protomers (Fig. S7B).

In conformation A, only five of the six LGF loops are bound to the shClpP2 surface, and two empty shClpP2 pockets are found between the ClpC1 protomers P6 and P2. The P1-LGF loop is likely in an undock position due to loss of the regional density in conformation A (Fig. 3C). In conformation B, all six of the LGF loops are mounted on the shClpP2 surface, and only one empty shClpP2 pocket is located between the P1 and P2 protomers of ClpC1. In both conformations, the protomer P3 of ClpC1 is bound most tightly because the P3-LGF loop is further stabilized by an upwardly extending C-terminus (C-ter′) of shClpP2 (Fig. 3D). At the other positions, the shClpP2 C-termini are folded outwardly away from the hydrophobic pocket and almost invisible (Fig. 3E). The weak anchor of the protomers P1, P5, and P6 may accommodate essential structural rearrangements of these ClpC1 protomers for the transition from conformation A to B.

### Substrate engagement by ClpC1 in the Clp protease machinery

Linear density representing the bound substrate of unfolded polypeptide (likely casein) was visualized in the central channel of ClpC1 in our determined ternary complex structures of ClpC1:shClpP1P2 (Fig. 1D). A poly-(Ala)_23_ chain was built into this substrate density in both conformations. The substrate unfolding and translocation are facilitated by pore loops that are generally conserved among AAA+ ATPases (Fig. S8A). The ClpC1 pore loops extend into the central channel and make extensive contacts with the substrate (Fig. S8B). The pore-1 loops are evenly distributed along the unfolded polypeptide substrate like a right-handed spiral staircase. Two adjacent pore-1 loops span two substrate residues in average, which is presumably correlated to the one-step translocation event driven by ATP hydrolysis.

**Fig 4:**
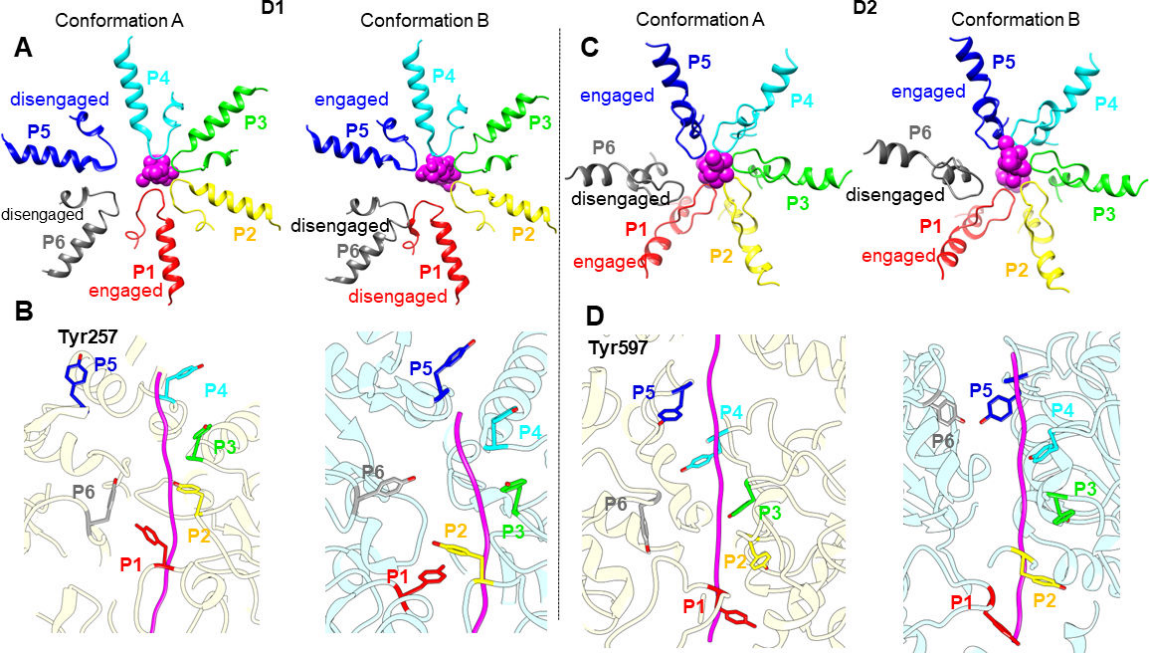
Substrate engagement by the ClpC1 pore loops. Comparison of conformation A and B reveals different substrate engagement states of the six ClpC1 protomers (P1–P6) in the D1 ring (left panel **A** and** B**) and the D2 ring (right panel **C** and D). The substrate engagement is shown in both the top views (upper panel **A** and **C**) and side views (bottom panel **B** and **D**). The polypeptide substrate is represented in magenta, and the six ClpC1 protomers are individually colored. The key tyrosine residues for substrate interaction, including Tyr257 of the D1 pore loop and Tyr597 of the D2 pore loop, are shown along the bound substrates, respectively.

In both conformations, the substrate densities are longer and better defined near the D2 domain in comparison with the D1 domain of ClpC1, which suggests the D2 domain of ClpC1 might hold the substrate more tightly (Fig. S8C). Indeed, four D1 pore-1 loops and five D2 pore-1 loops are found bound to the substrate backbone in either conformation. However, the hexameric ClpC1 in the two identified conformations is engaged with the substrate in distinct mode. In both conformations, the D1 and D2 pore-1 loops of the protomers P2, P3, and P4 stay in touch with the substrate, while the protomer P6 remains uncontacted. Differently, the P1 D1 pore-1 loop is bound in conformation A but unbound in conformation B, whereas the P5 D1 pore-1 loop switches from the disengaged state in conformation A to the engaged state in conformation B (Fig. 4A). The D2 pore-1 loops of the protomers P1 and P5 are kept engaged with the substrate in both conformations (Fig. 4C). Although the protomer P6 is not engaged with the substrate in both conformations, the P6 D2 pore-1 loop takes an upward shift of ~15.4 Å (distance between the CA atoms of Tyr597) along the substrate in conformation B (Fig. S8D). The different D1 engagement states of the protomers P1 and P5 may facilitate substrate translocation through the central channel of the D1 ATPase ring. The upward movement of the P6 D2 domain may represent a pre-state ready for the next step of substrate engagement in the D2 ATPase ring.

### Nucleotide states of the ClpC1 ATPase domains

**Fig 5:**
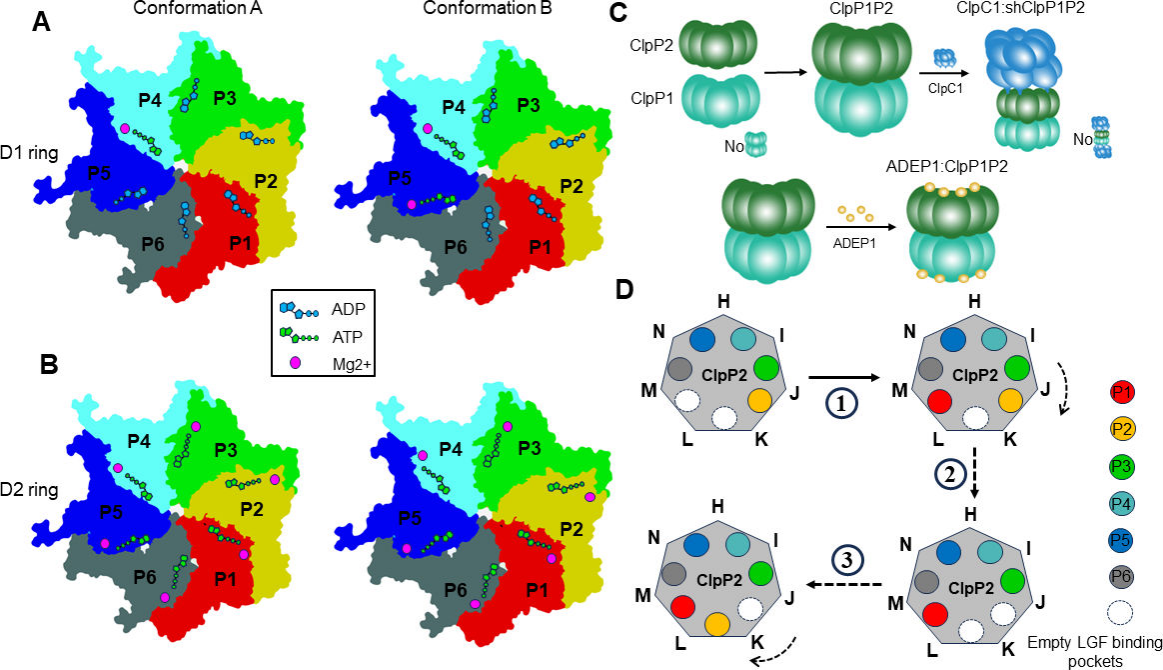
Schematic of the ClpC1 nucleotide states in the two conformations and hypothetical mechanistic model for the substrate degradation by the ClpC1:shClpP1P2 machinery. (A–B) Schematic representations of the nucleotide states in the D1 ring (**A**) and the D2 ring (**B**) of ClpC1 in conformation A and conformation B. (**C)** Assembly of the functional Clp system in *Streptomyces*.** (D)** A proposed pathway for the ClpC1 rotation on the shClpP2 surface that is associated with the substrate transportation from the ClpC1 D1/D2 rings to the shClpP1P2 degradation chamber. The shClpP2 protomers are labeled clockwise from H to N according to their chain positions.

The ClpC1 protomer possesses two ATP hydrolysis domains in tandem. The nucleotide states (ATP as pre-hydrolysis, ADP as post-hydrolysis, and apo for empty pocket) for each D1 or D2 domain of ClpC1 were individually determined in both identified conformations based on the ligand densities, coordination with magnesium ions, and vicinal arginine (Arg) fingers (Fig. S9A and B). The γ-phosphate of the bound ATP in the catalytic center is usually stabilized by the Arg fingers extruding from the clockwise adjacent protomer of the ATPase. Notably, only ATP molecules are bound in the D2 domains of ClpC1 for both conformation A and B. In contrast, ADP is the major form within the ClpC1 D1 domains, except that ATP is found in the D1-P4 position of conformation A and in the D1-P4 and D1-P5 positions of conformation B (Fig. 5A and B). To our surprise, the D1 domains of the protomers P2 and P3 are in the ADP state but with engaged Arg fingers (R336-R337) nearby (Fig. S9C and D). Both the protomers P2 and P3 are engaged with the substrate and nearly show no structural difference between two conformations.

The ClpC1 D2 domains are trapped in the pre-hydrolysis states, whereas both pre- and post-hydrolysis states are observed in the ClpC1 D1 domains. Thus, ATP hydrolysis by the D1 and D2 ATPase rings seems not closely coupled in ClpC1. ATP hydrolysis might be initiated by the D1 ring, likely at the P3 position where the ClpC1 protomer is tethered by the C-terminus of shClpP2. Similar to other AAA+ ATPases, the substrate is likely translocated by ClpC1 following a rotational mechanism. Because ATP states are only at the D1 P4/P5 positions, ATP hydrolysis conducted by the ClpC1 D1 domains is probably in the clockwise direction (viewed from the top of the D1 ring), which is similar to most other AAA+ ATPases. However, the energy generated from ATP hydrolysis is not immediately released but stored in the protomers P2 and P3 that are still engaged with the substrate. The stored energy might be sequentially released later from the ATPase protomer concurrently with structural rearrangement as proceeding with substrate translocation.

## DISCUSSION

The Clp protease system is a good target for the development of antibacterial therapy because of its important role in maintaining intracellular proteostasis and regulating bacterial virulence. *Streptomyces* can produce a variety of natural products with significant bioactivities. The Clp system in *Streptomyces* is sophisticated and involves five redundant ClpP homologs. In this study, we focused on the house-keeping Clp proteins shClpP1 and shClpP2 from *S. hawaiiensis* and revealed the assembly of the hetero-tetradecameric degradation chamber of shClpP1P2 and the AAA+ unfoldase ClpC1. Two distinct conformations of the substrate-bound ClpC1:shClpP1P2 have been identified representing two sequential states in a substrate translocation event. The structure of shClpP1P2 in complex with ADEP1 was also determined (Fig. 5C).

Different from some other Clp proteins, shClpP1 and shClpP2 from *S. hawaiiensis* form a hetero-tetradecameric complex. Our results show that shClpP1 can only form a heptameric ring structure (Fig. S1A), and the handle domain specialty of shClpP1 may prohibit it from forming a homo-tetradecameric structure. During our cryo-EM image process of the shClpP1P2 reconstruction, we did not observe any portion of the shClpP1 or shClpP2 oligomers. It suggests that shClpP2 is unlikely to form a homo-tetradecamer. However, if shClpP1 and shClpP2 could form hetero-tetradecamers with the other homologs, shClpP3/P4/P5 from the same host remains to be investigated.

The hydrophobic interaction between the diverse I/LGF loops of the AAA+ ATPases and the same ClpP hydrophobic pocket accounts for the constitution of various ATPase-ClpP protease systems. The apical hydrophobic pockets of shClpP1 and shClpP2 in the intermolecular clefts are similar in amino acid compositions but slightly different in the volume size. The pocket volume of the shClpP2 hydrophobic pocket (~774.17 Å^3^) is larger than that of the shClpP1 hydrophobic pocket (~512.32 Å^3^). Therefore, the shClpP2 hydrophobic pocket could accommodate the LGF loop easier than the shClpP1 hydrophobic pocket. In addition, the C-terminus of shClpP2, which could interact with the LGF loop stem of ClpC1, is missing in shClpP1. This may explain why the shClpP1P2 chamber is only single capped by ClpC1 on the shClpP2 surface.

ADEP could accelerate the ATPase-dependent degradation of natural substrates in *Streptomyces* (13). Recent biochemical studies of shClpP1P2 suggested that ADEP may only bind to shClpP1 without interfering the attachment of ClpC1 to shClpP2 (13). However, our structure showed the ADEP1 molecules are bound by both shClpP1 and shClpP2. The average density height of ADEP1 bound in the shClpP1 hydrophobic pocket is higher than that of ADEP1 bound by shClpP2. Normalized density of the ligand against the protein backbone could be used to estimate the ligand occupancy. The calculated ADEP1 occupancy in the shClpP1 hydrophobic pocket (~93.94%) is higher than that in the shClpP2 hydrophobic pocket (~70.03%). It suggests that the shClpP1 hydrophobic pocket is favored by the ADEP1 molecule. The spacious shClpP2 pocket may not be ideal for stabilizing the bound ligand such as ADEP1. The interactive residues for ADEP1 and the LGF loop are partially shared in the shClpP hydrophobic pockets, such as Tyr76, Tyr78, Tyr98′ of shClpP1 and Ser94, Tyr96, Tyr116′ of shClpP2. It indicates that binding of ADEP1 may affect the proteolytic function of the ClpP chamber by interfering the interaction between ClpP and the ATPase chaperone or by mimicking the LGF loop docking. These interactive residues are highly conserved among ClpP homologs in different species (Fig. S5C). A previous biochemical study of ClpP in *B. subtilis* showed that the caseinolytic activity was greatly diminished in the ClpP mutants of Y62 and F82′ (equivalent to Y78, Y98′ in shClpP1 and Y96, Y116′ in shClpP2) that are defective in binding ADEPs (28).

Similar to the reported ClpXP and ClpAP systems, the flexibility of the LGF loops of ClpC1 provides the structural plasticity necessary for overcoming the hexamer-heptamer symmetry mismatch between the ATPase rings and the ClpP proteolytic chamber. It also ensures the asymmetric ClpC1 hexamer could move in a moderate range that is required for continuous ATP hydrolysis and the polypeptide substrate translocation through the central channel. The ClpC1 protomers at the seam positions contribute most to the structural rearrangement of the ClpC1 ring while processing the substrate. During the transition from conformation A to B, the protomers P2, P3, and P4 remain anchored onto the shClpP2 surface and keep in contact with the substrate for stabilization. The seam protomer P6 undergoes major conformational changes, which spread into its neighborhood of the protomers P1 and P5. The protomer P1 dissociates its D1 domain from low position on the substrate and docks its LGF loop of the D2 domain into a nearby empty hydrophobic pocket of shClpP2. At the meanwhile, the protomer P5 takes over the substrate by moving its D1 domain toward the top position of the spiral staircase (Fig. 4B and D). In a continuous translocation event, the substrate could be gripped downward “hand over hand” by ClpC1 and propelled toward the proteolytic ClpP chamber (Fig. 5D).

For the AAA+ ATPases containing tandem ATPase domains, the ATPase activity of the D2 ring is proven more critical for substrate translocation than that of the D1 ring (34). It is supported by the fact that the D2 ATPase domains share high similarity in sequence with the AAA+ ATPases containing only one ATPase domain such as ClpX. However, how the D1 and D2 rings coordinate ATP hydrolysis is still uncertain. Our structures showed that ATP molecules were only consumed by the D1 ring of ClpC1. The D2 catalytic activity of ClpC1 is restricted by the Walker B mutations and the interaction with the shClpP2 layer. In contrast, the detrimental effect of the Walker B mutation in the D1 domain of ClpC1 is compensated by the MD mutation. Therefore, the D1 ATPase ring is likely more functional in ATP hydrolysis compared with the D2 ATPase ring of ClpC1. Our complex structures might be trapped in the states that the D1 ATPase ring plays a dominant role in ATP hydrolysis for substrate unfolding and translocation. It suggests that the D1 and D2 domains from the same or neighboring protomers may not necessarily hydrolyze ATP in tandem while processing the polypeptide substrate. The ATP hydrolysis activity of the D1 ATPase ring could be independent of the D2 ATPase function. This finding is in agreement with a previous ClpB study (34).

ATP binding, hydrolysis, and release associated with conformational changes are correlated to different substrate engagement states of the ATPases. In general, the ADP-bound and apo states are mostly linked to the substrate-disengaged protomers of the hexameric ATPase. However, our structures indicate that the ClpC1 P2 and P3 D1 domains are still in the ADP-bound forms while being engaged with the substrate. Thus, the energy generated from ATP hydrolysis could be stored in these engaged protomers until conformational change is desired for one-step translocation process. Although conformational rearrangement lags a few steps behind ATP hydrolysis, both events could still be sequentially conducted following a clockwise rotational pattern along the ClpC1 hexameric ring.

In summary, our results provide a new perspective on the conformational changes of substrate translocation during ATP hydrolysis and protein degradation in the Clp system. Further elucidation of this dynamic and highly flexible protein degradation mechanism is needed in the future. Overuse of commonly used antibiotics that target protein, nucleic acid synthesis, and cell wall assembly has led to the emergence of multidrug-resistant pathogens. The ClpC1P1P2 protease is an attractive target for developing antimicrobial drugs due to its important role in maintaining proteostasis and counteracting host-induced stress. Bacterial proteolysis targeting chimeras have been designed based on the complex of ClpC1:ClpP1P2 to improve the effectiveness of drugs targeting the Clp protease system (35, 36) and provide an attractive technology platform for the development of next-generation antibiotics. Our high-resolution structures may provide basis for the strategy enhancement fighting against bacterial multidrug resistance.

## MATERIALS AND METHODS

### Plasmids and constructs

The PCR amplifications of *shclpP1* (*orf* CEB94_14110), *shclpP2* (*orf* CEB94_14105), and *clpC1* (*orf* CEB94_23085) were carried out using the genomic DNA of *S. hawaiiensis* NRRL 15010 as the template. The DNA fragments were cloned into between the *Nde*I and *Xho*I sites of pET28a+, resulting in a hexa-His tag followed by a TEV cleavage site at the N-terminus. Point mutations were introduced using the Quikchange mutagenesis method (37).

### Protein purification of shClpP1, shClpP2, and ClpC1

The recombinant plasmids were transformed into *E. coli* BL21(DE3) and cultured in LB medium at 37°C until OD_600_ reached 0.6. The incubation temperature was decreased to 18°C. The desired proteins were then induced for a 16-h expression by adding 4 mM isopropyl-β-d-thiogalactoside. Cells were lysed in the buffer containing 50 mM HEPES, 300 mM KCl, 10% glycerol, and pH 7.5, and the lysate was harvested after centrifugation at the speed of 17,000 × *g* and 4°C for 30 min. The supernatant was then loaded onto the Ni^2+^-NTA gravity column (Genscript, China) for purification of our target recombinant proteins. After column wash in the buffer containing 50 mM HEPES, 300 mM KCl, 10% glycerol, 50 mM imidazole, and pH 7.5, the target proteins were eluted in the buffer containing 50 mM HEPES, 300 mM KCl, 10% glycerol, pH 7.5, and 100 mM imidazole. Fractions containing the target protein were pooled and subjected to size exclusion chromatography (SEC) using a Superdex 200 Increase 16/600 (Cytiva, USA) column in the SEC buffer (20 mM HEPES, 150 mM KCl, 5% glycerol, pH 7.5). For purification of the ClpC1 protein, 20 mM MgCl_2_ and 10 mM ATP were supplemented in the buffer at each step to ensure formation of the hexamers.

### Preparation of the shClpP1P2 binary complex and the ClpC1:shClpP1P2 ternary complex

A stable shClpP1P2 complex was obtained by mixing individually purified shClpP1 and shClpP2 at 1:1 molar ratio followed by 1 h incubation at room temperature and passing through the SEC for fraction collection. For the preparation of ClpC1:shClpP1P2, on the basis of the binary complex prepared in the previous step, an equal amount of ClpC1 was added and incubated at room temperature for 1 h. Both complexes were diluted to 2–5 mg/mL in the SEC buffer for cryo-EM analysis.

### Cryo-EM sample preparation and data acquisition

An amount of 3 µL of protein sample (shClpP1, or shClpP1P2, or ClpC1:shClpP1P2) at a concentration of 2 mg/mL was applied to the EM grids (Quantifoil Cu R1.2/1.3, 300 mesh) that had been glow discharged for 50 s using Pelco easiGlow. The samples were plunged frozen in liquid ethane using a ThermoFisher Vitrobot with the settings of temperature 8°C, relative humidity 100%, blot force 3, and blot time 3 s. Cryo-EM data collection was performed on a ThermoFisher Scientific 300 kV TEM Titan Krios equipped with Gatan K3 direct electron detector, using the EPU software (ThermoFisher) for automated data acquisition (Table 1). Data were collected at a defocus range of −1.1 to −1.5 µm and at a nominal magnification of 105,000×, which resulted in a calibrated pixel size of 0.42 Å/pixel. Micrographs were recorded as movie stacks with an electron dose of 50 e^−^/ Å^2^ fractionated into a total of 50 frames.

### Image processing, reconstruction, and model refinement

All image processing was performed using Cryosparc v4.0 (38). For each data set, patch motion correction on movie-stacks and patch CTF estimation on dose-weighted micrographs were applied. The binned micrographs with a final pixel size of 0.84 Å/pixel were used for data processing. Particles were auto-picked using the deep learning method of Topaz (39). After several rounds of non-reference 2D classification, the selected good particles were subjected to *ab initio* model reconstructions followed by heterogeneous refinement and 3D classifications. Non-uniform refinement was done for each group of reconstructions. The resolutions of final reconstructions were estimated using gold standard with a 0.143 FSC criterion. Local resolutions were also calculated. To improve map quality of the ClpC1:shClpP1P2 complex, local refinement focused on the ClpC1 or shClpP1P2 region was further performed using the corresponding mask. The regional maps of ClpC1 and shClpP1P2 were finally combined using Phenix (40) for easy interpretation of the results. The atomic models were built in Coot (41) based on the initial protomer models predicted by AlphaFold 2 (42). Real space refinement and validation of the models were done using Phenix.

### Sequence alignment and structure analysis

Protein sequence alignment was done using ClustalW (43) and ESPript (44). The pocket cavity was calculated using ProteinsPlus (45). The molecular interactions were analyzed using online servers PISA (46) and PLIP (47). Pymol (Schrödinger, LLC) or UCSF ChimeraX (48) was used for other structural analysis and figure preparation.

## Data Availability

Cryo-EM maps of this study have been deposited in the Electron Microscope Data Bank (EMDB) with the accession codes EMD-38497, EMD-38537, EMD-38536, and EMD-38535. The atomic coordinates and structure factors for the structures determined in this study have been deposited in the Protein Data Bank under the accession codes 8XN4, 8XOP, 8XOO, and 8XON.

## SUPPLEMENTAL MATERIAL

Supplemental Figures and Video legend mbio.00031-24-s0001.docx Fig. S1 to S9 and legend to Video S1.

Video S1 mbio.00031-24-s0002.MP4 Conformational transition of the ClpC1:shClpP1P2 machinery.

ASM does not own the copyrights to Supplemental Material that may be linked to, or accessed through, an article. The authors have granted ASM a non-exclusive, world-wide license to publish the Supplemental Material files. Please contact the corresponding author directly for reuse.
