# Supplementary material for: Structural insights into the Clp protein degradation machinery
Source: mBio. 2024 Mar 19;15(4):e00031-24. doi: 10.1128/mbio.00031-24 (PMC11005422; doi:10.1128/mbio.00031-24)
Supplement: Supplemental Figures and Video legend — Fig. S1 to S9 and legend to Video S1. [file mbio.00031-24-s0001.docx]

**Supplementary Information for:**

**Structural insights into the Clp protein degradation machinery**

Xiaolong Xu^a,b^, Yanhui Wang^a,b^, Wei Huang^a,b^, Danyang Li^c^, Zixin Deng^b^, Feng Long^a,b,#^

^a^Department of neurosurgery, Zhongnan Hospital of Wuhan University, School of Pharmaceutical Sciences, Wuhan University, Wuhan 430071, China

^b^Ministry of Education Key Laboratory of Combinatorial Biosynthesis and Drug Discovery, School of Pharmaceutical Sciences, Wuhan University, Wuhan 430071, China

^c^Cryo-EM center and the core facility of Wuhan University, Wuhan 430071, China

#Address correspondence to Feng Long, [longfe@whu.edu.cn](mailto:longfe@whu.edu.cn)

**Supplementary Information includes:**

- Figs. S1 to S9 and legends
- Supplementary Video 1 legend


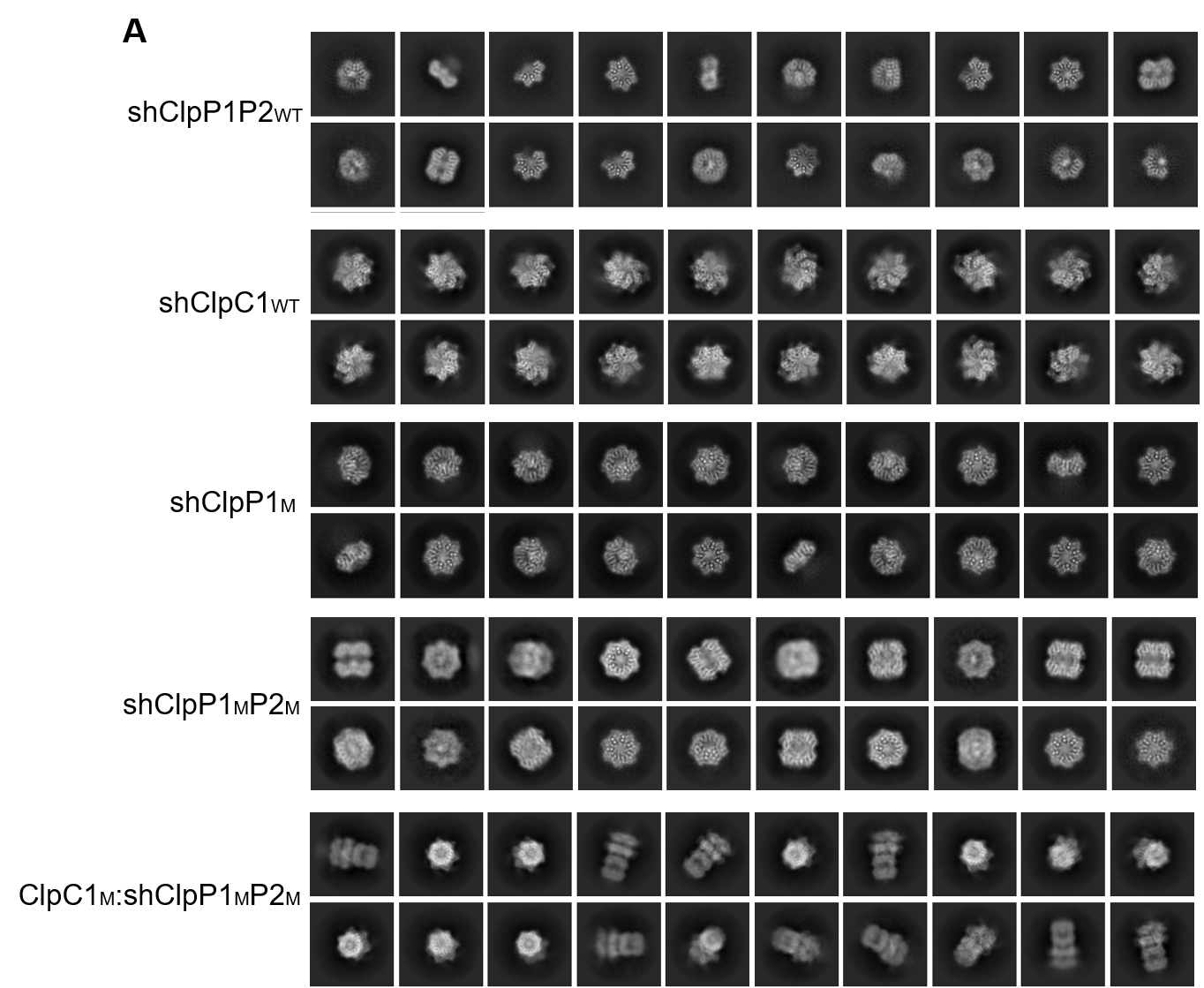


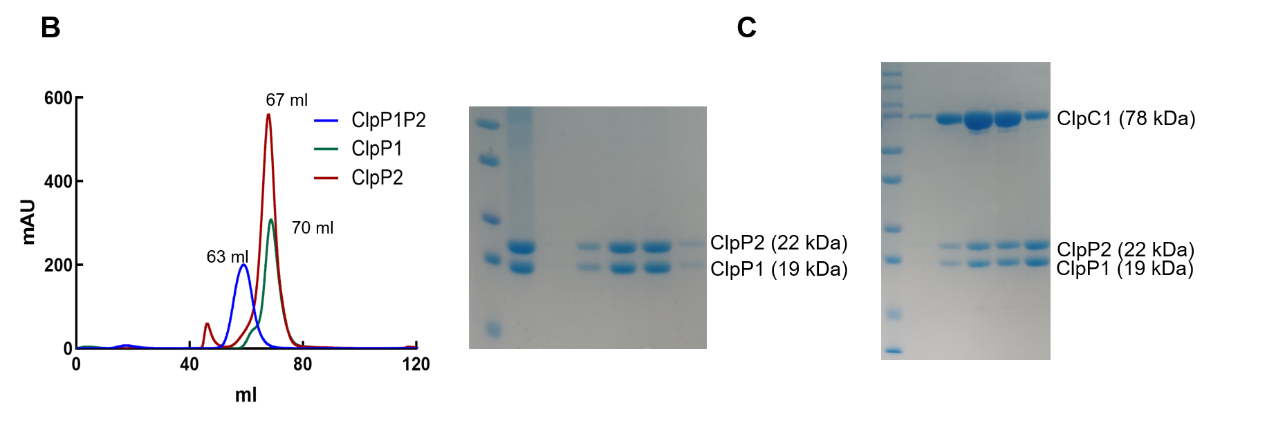


**Fig. S1 Sample preparation of the shClpP1P2 and ClpC1:shClpP1P2 complexes. A,** Representative class averages from the non-reference 2D classification of different protein samples that were used for cryo-EM analysis. The purified proteins with the designed mutations (shClpP1_M_, shClpP2_M_, ClpC1_M_) are more homogeneous than the purified wild-type proteins (shClpP1_WT_, shClpP2_WT_, ClpC1_WT_). The mutations include S113A in shClpP1_M_, S131A in shclpP2_M_, and E284A/E622A/F440A in ClpC1_M_. **B,** The size exclusion chromatography profiles of the purified shClpP1, shClpP2 and shClpP1P2 proteins. The elution peaks are labeled for each sample accordingly. The protein elution fractions of shClpP1P2 were examined by the SDS-PAGE analysis. **C,** The SDS-PAGE analysis of the prepared ternary complex of ClpC1:shClpP1P2.


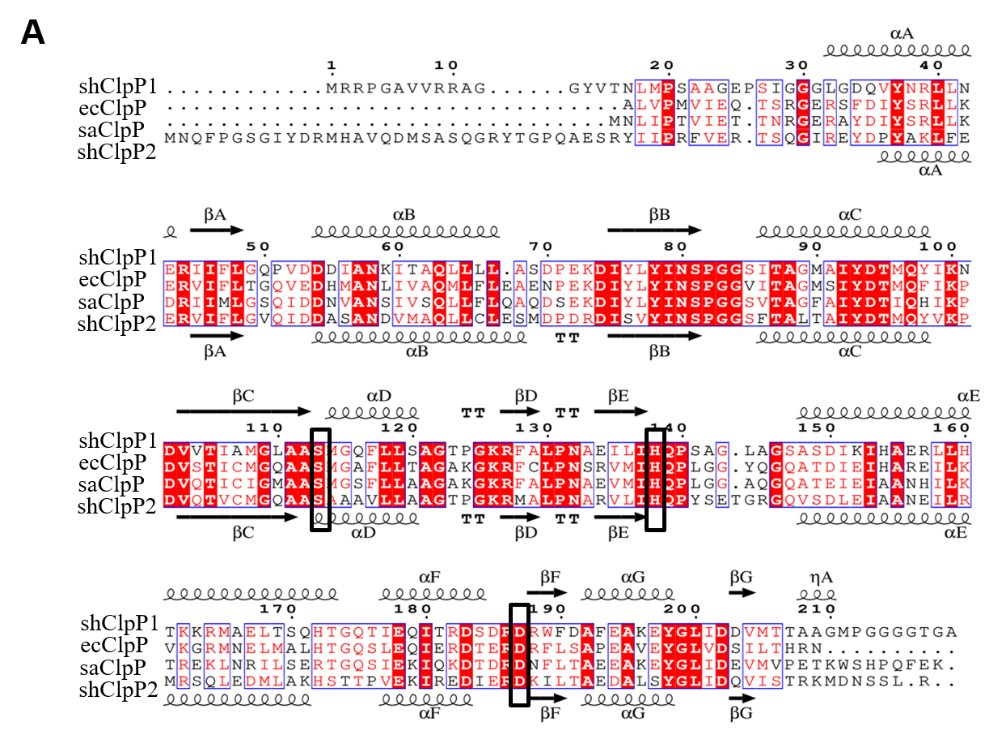


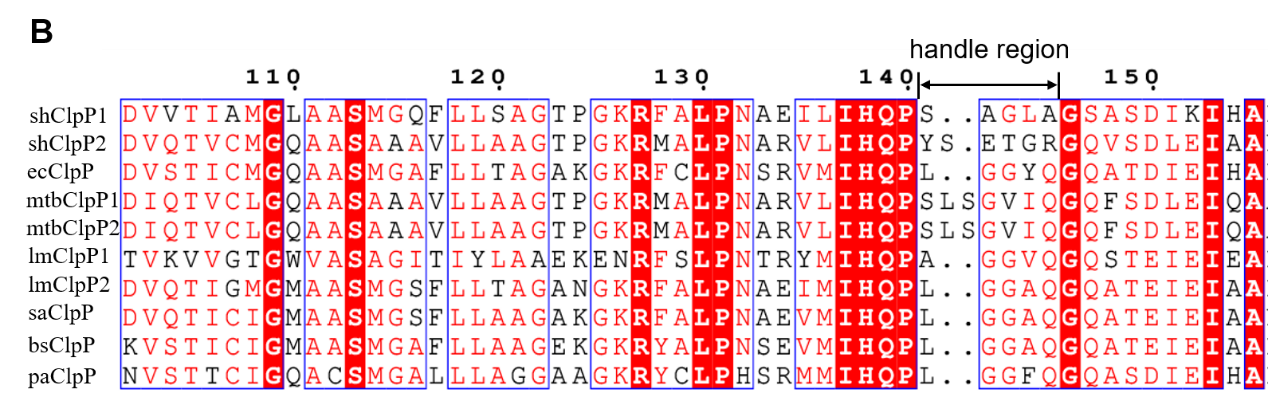


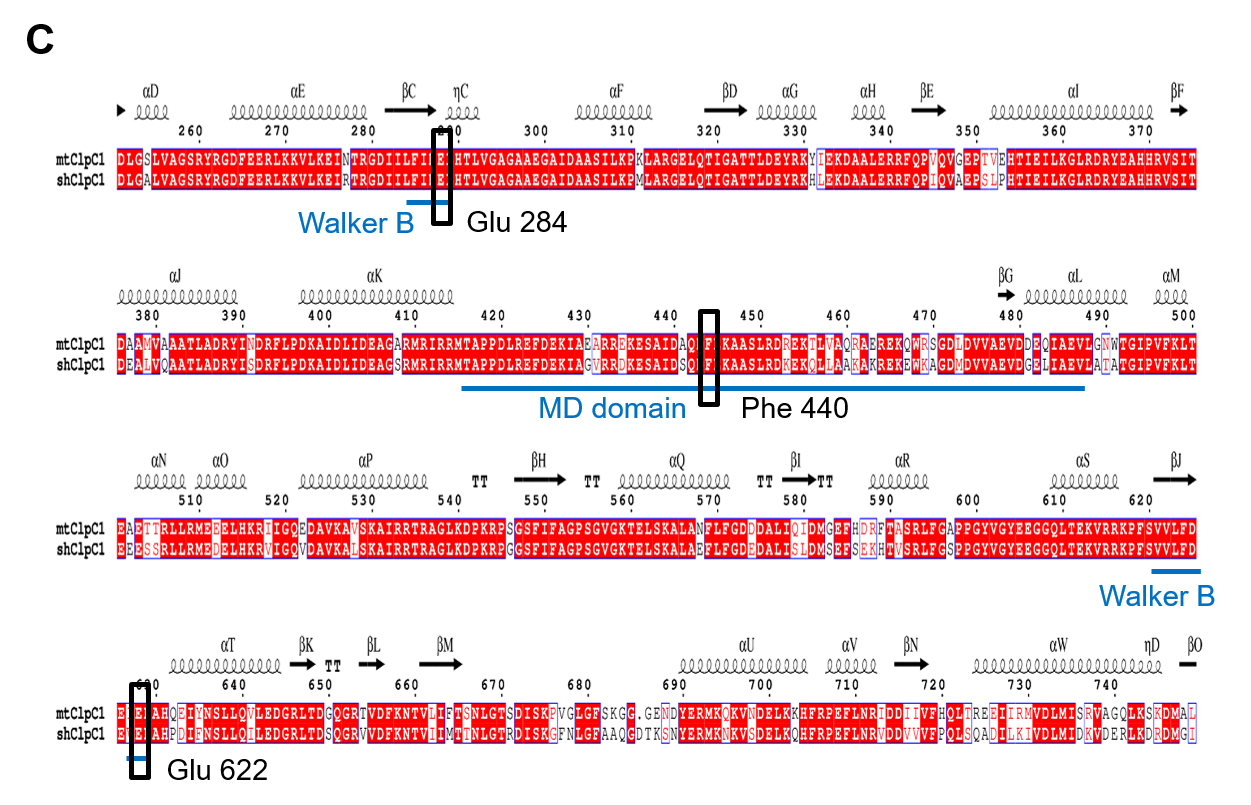


**Fig. S2 Protein sequence alignments of shClpP1, shClpP2，ClpC1 from** ***Streptomyces hawaiiensis* and their homologues. A,** The sequence alignment of shClpP1, shClpP2 and their two homologues. Three conserved catalytic residues are indicated in the black rectangles. **B,** The sequence alignment of the handle domains of shClpP1, shClpP2 and the homologues. **C,** The partial sequence comparison between two ClpC1 homologous proteins. The mutation sites that could stabilize the protein complexes are indicated in the Walker-B (E284A/E622A) and MD (F440A) domains. sh: *Streptomyces hawaiiensis*; ec: *Escherichia coli*; sa: *Staphylococcus aureus*; mtb/mt: *Mycobacterium tuberculosis*; lm: *Listeria monocytogenes*; bs: *Bacillus subtilis*; pa: *Pseudomonas aeruginosa*.


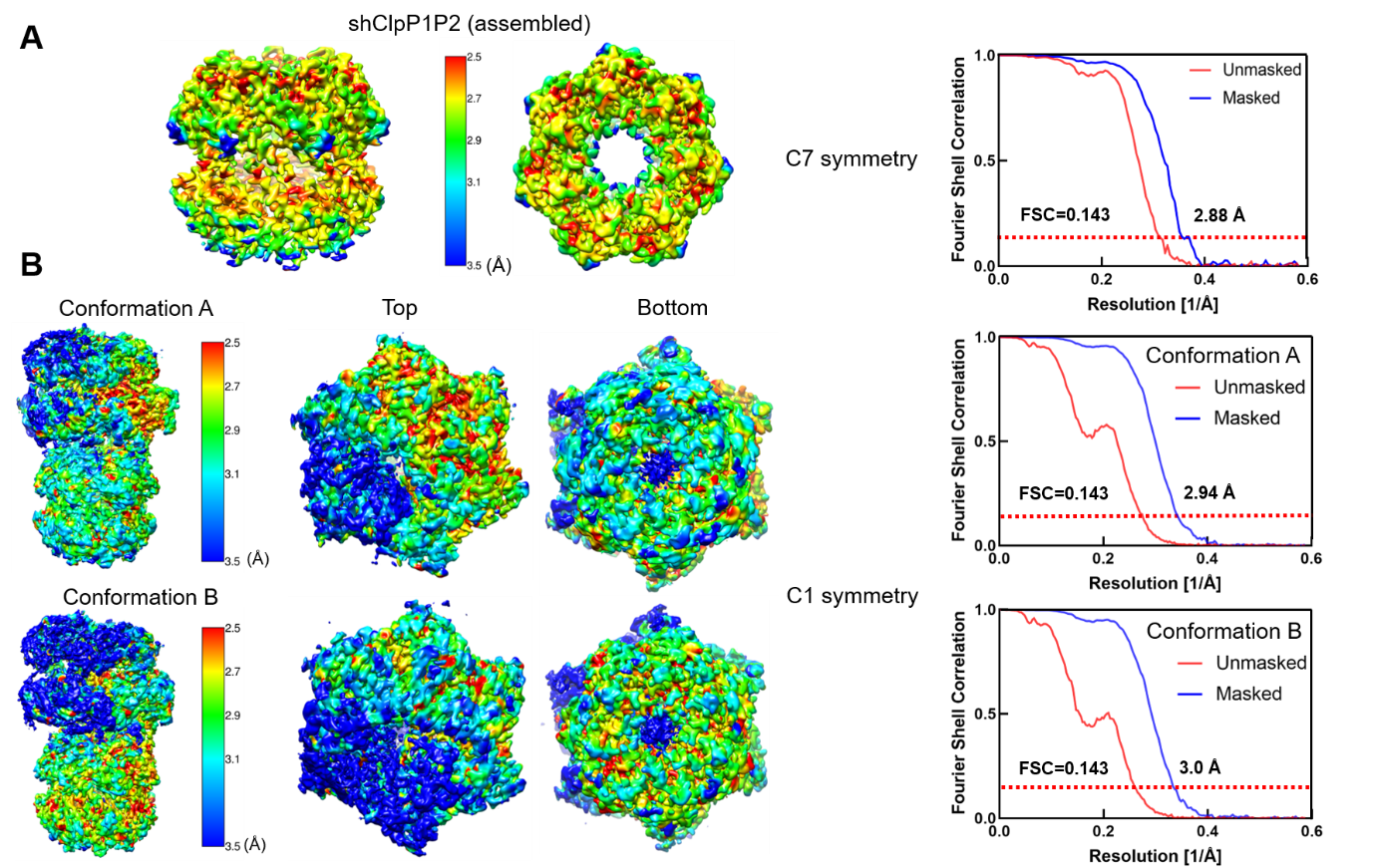


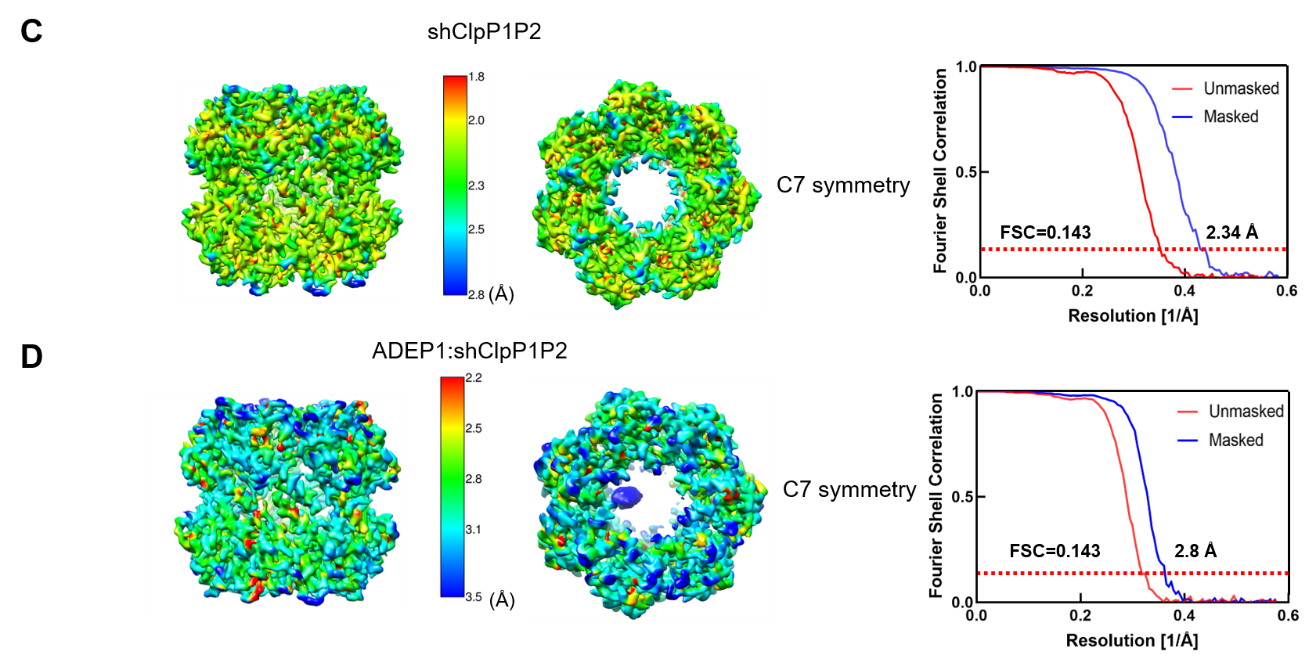


**Fig. S3 Resolution estimation of the cryo-EM reconstructions of shClpP1P2, ClpC1:shClpP1P2 and ADEP1:shClpP1P2. A,** Cryo-EM reconstruction of the assembled shClpP1P2 complex with an imposed C7 symmetry. **B,** Cryo-EM reconstructions of the conformation A (C1 symmetry) and conformation B (C1 symmetry) of ClpC1:shClpP1ClpP2. **C,** Cryo-EM reconstruction of shClpP1P2 (C7 symmetry) from the dataset collected for the assembled ClpC1:shClpP1ClpP2 sample. **D,** Cryo-EM reconstruction of the assembled shClpP1P2 in complex with ADEP1 with an imposed C7 symmetry. The cryo-EM maps are colored according to the estimated local resolutions. The Fourier shell correlation (FSC) coefficients are plotted for the masked and unmasked cryo-EM reconstructions. The overall resolutions of the masked reconstructions were estimated using gold standard with a FSC 0.143 cutoff and indicated near the FSC curves.


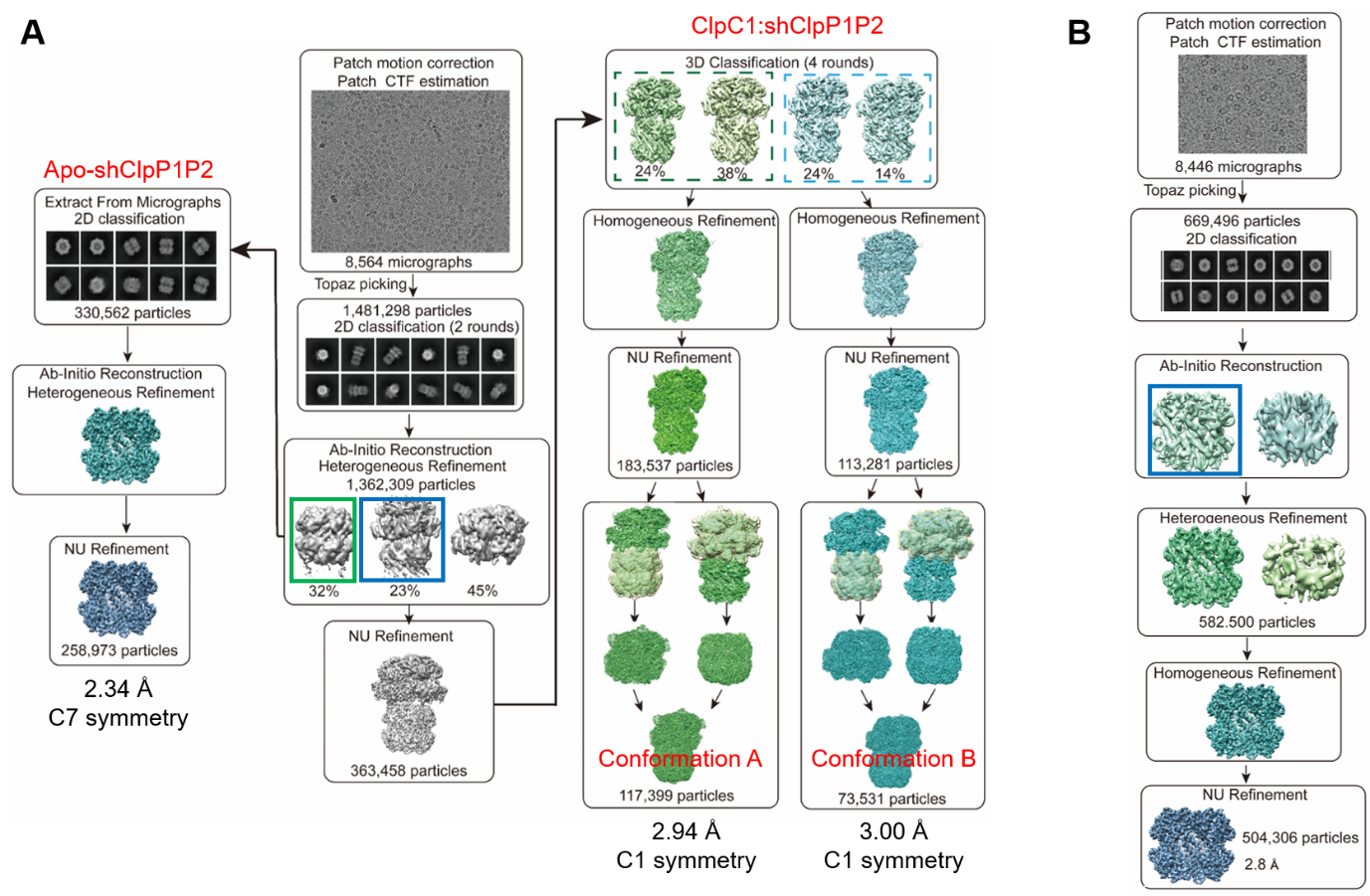


**Fig. S4 Workflows for the cryo-EM structural determination of the Clp protease complexes from *Streptomyces hawaiiensis*.** **A,** Flowchart of the cryo-EM data processing for the assembled ClpC1:shClpP1P2. The reconstructions of two conformations (conformation A and conformation B) of ClpC1:shClpP1P2, and the reconstruction of apo-shClpP1P2 were finally obtained. **B,** Flowchart of the cryo-EM data processing for the assembled shClpP1P2 in complex withADEP1.


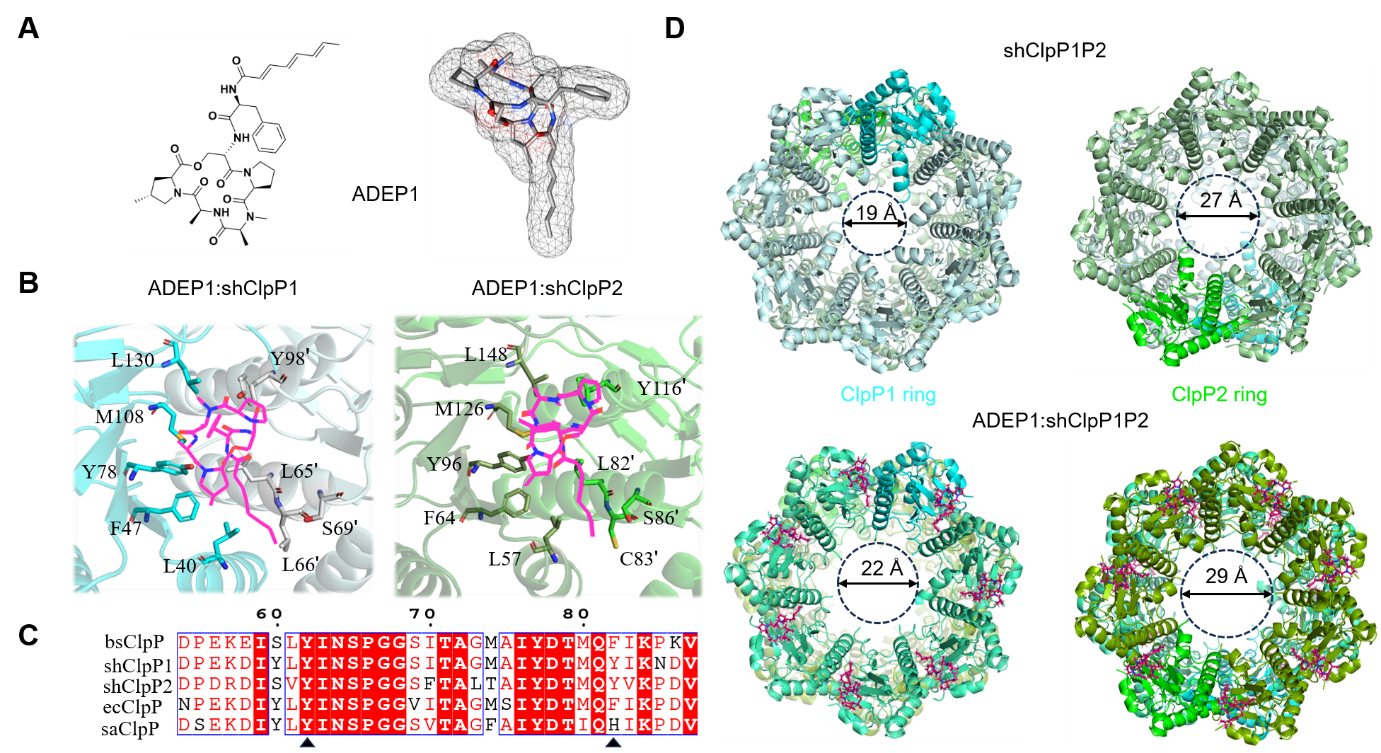


**Fig. S5 Binding of ADEP1 in the shClpP1P2 complex. A,** Chemical structure of the natural product ADEP1, and a representative ADEP1 model fit in the EM density that is segmented from ADEP1:shClpP1P2 and contoured at a 3σ level. **B,** Interactions of shClpP1 and shClpP2 with the bound ADEP1. **C,** Sequence alignment of ClpPs indicates the conservation of critical ADEP1 interactive residues. Two residues equivalent to Y62 and F82 in bsClpP are marked with black triangles. sh: *Streptomyces hawaiiensis*; ec: *Escherichia coli*; sa: *Staphylococcus aureus*; bs: *Bacillus subtilis*. **D,** Slight dilation of the shClpP1P2 central channel upon binding of ADEP1. The heptameric rings of shClpP1 (left) and shClpP2 (right) are shown for shClpP1P2 (upper panel) and ADEP1:shClpP1P2 (bottom panel), respectively. The approximate internal diameters of the central channels are indicated within the dashed circles. The bound ADEP1 molecules are shown as magenta sticks.


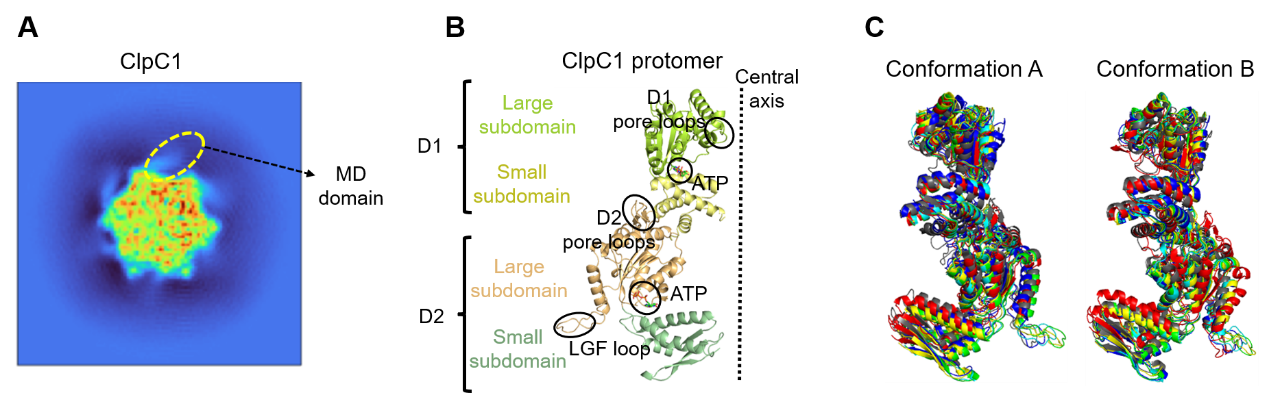


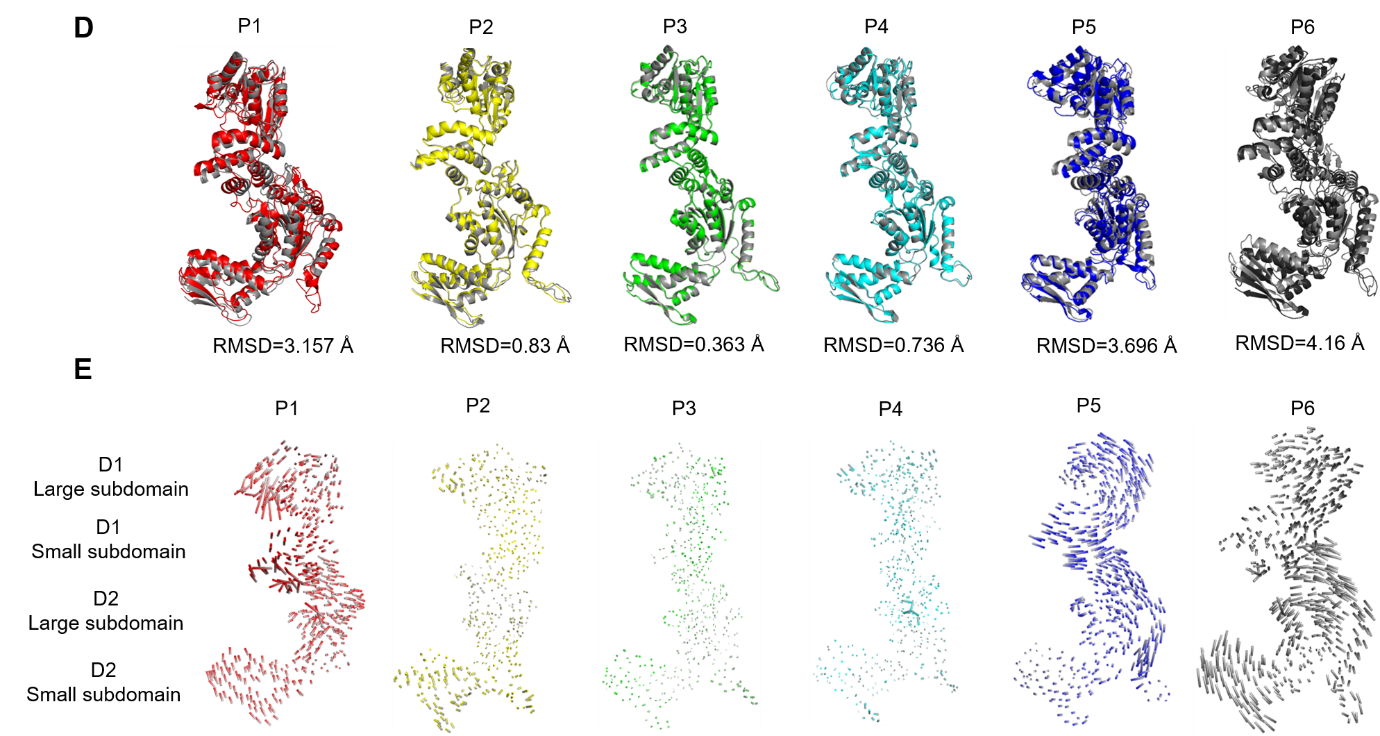


**Fig. S6 Structural flexibility of the ClpC1 protomers. A,** Projection view of the ClpC1:shClpP1P2 reconstruction adopted from Cryosparc shows the extended MD domains of ClpC1. **B,** Structural characteristics of a ClpC1 protomer. The subdomains and some critical motifs are labeled. Location of the central axis of the hexameric ClpC1 is indicated by a dashed line. **C,** Structural superimposition of the six ClpC1 protomers within the hexameric ClpC1 structure from conformation A (left) and conformation B (right). **D,** Structural superimposition of the paired ClpC1 protomers from two conformations are individually performed. The RMSD values (between the paired 563 Cα atoms) are calculated, indicating the largest structural differences of ClpC1 at the P1, P5 and P6 positions. **E,** Movement of Cα atoms is tracked when the ClpC1 protomer morphs from conformation A to B, as indicated by each color from light to dark. The significant movement is observed in the subdomains of ClpC1 at the P1, P5 and P6 positions.


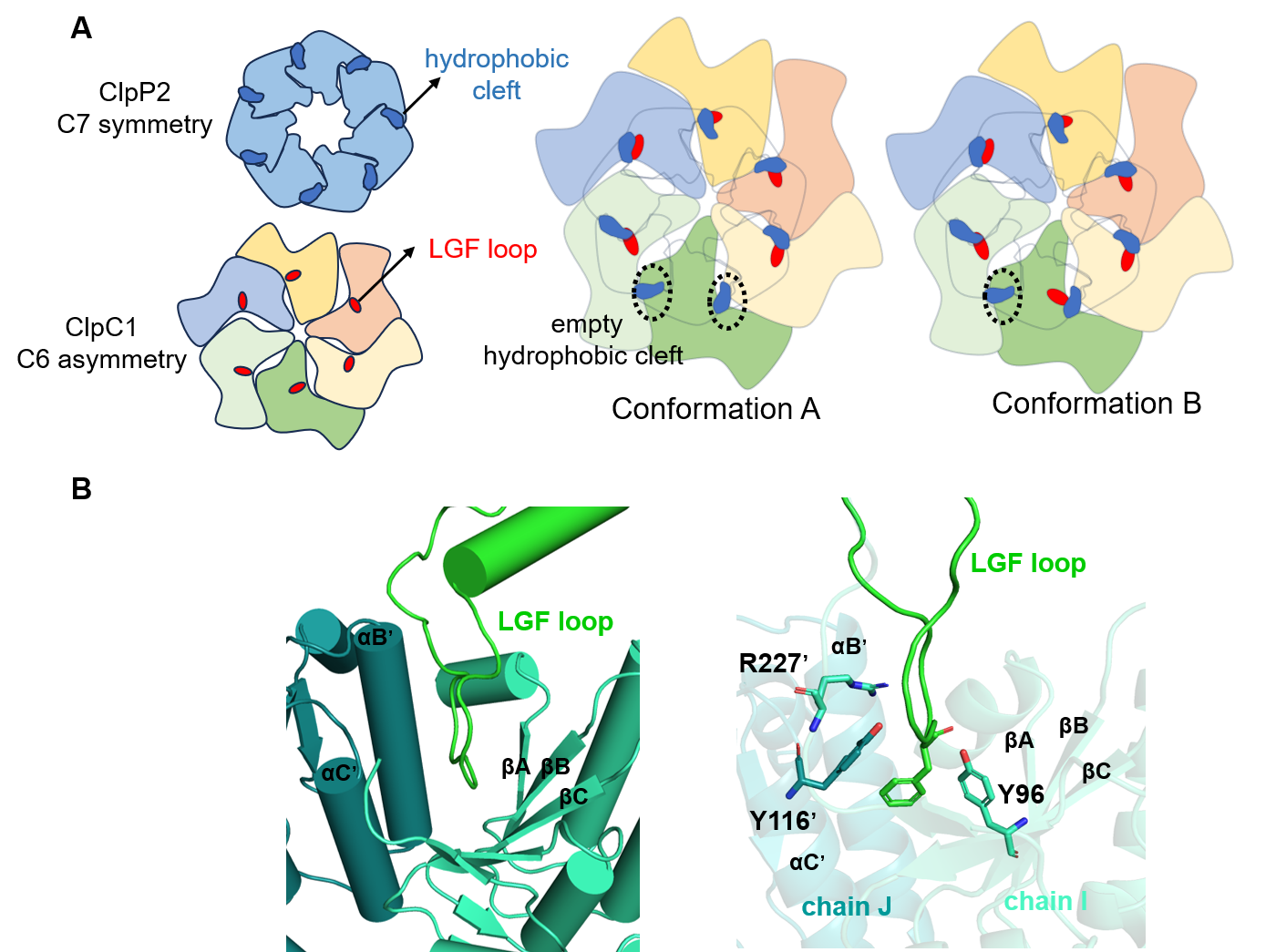


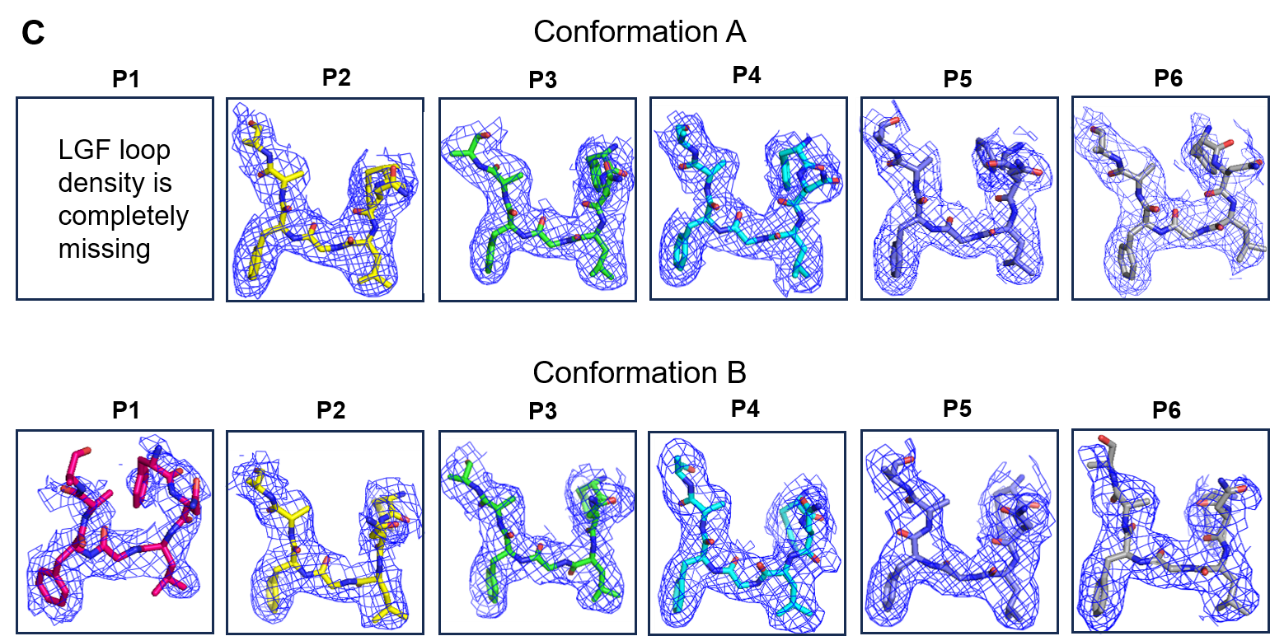


**Fig. S7 Interaction between ClpC1 and shClpP2. A,** Diagrams of the interaction between the ClpC1 hexamer and the shClpP2 heptamer in conformation A and conformation B. The LGF loops of ClpC1 are bound in the hydrophobic clefts of shClpP2. The unoccupied shClpP2 hydrophobic grooves are circled with dashed lines in both conformations. **B,** Interaction with the bound LGF loop (cyan) of ClpC1 in a hydrophobic pocket formed by two adjacent shClpP2 protomers (olive green and green). The LGF loop of ClpC1 is stabilized by forming hydrogen bonds with Y96 of shClpP2 (chain I), and Y116’ and R227’ of the clockwise adjacent shClpP2’ (chain J). **C,** All the bound LGF loops of the ClpC1 protomers in conformation A and conformation B are shown in the cryo-EM density maps contoured at 3σ, respectively.


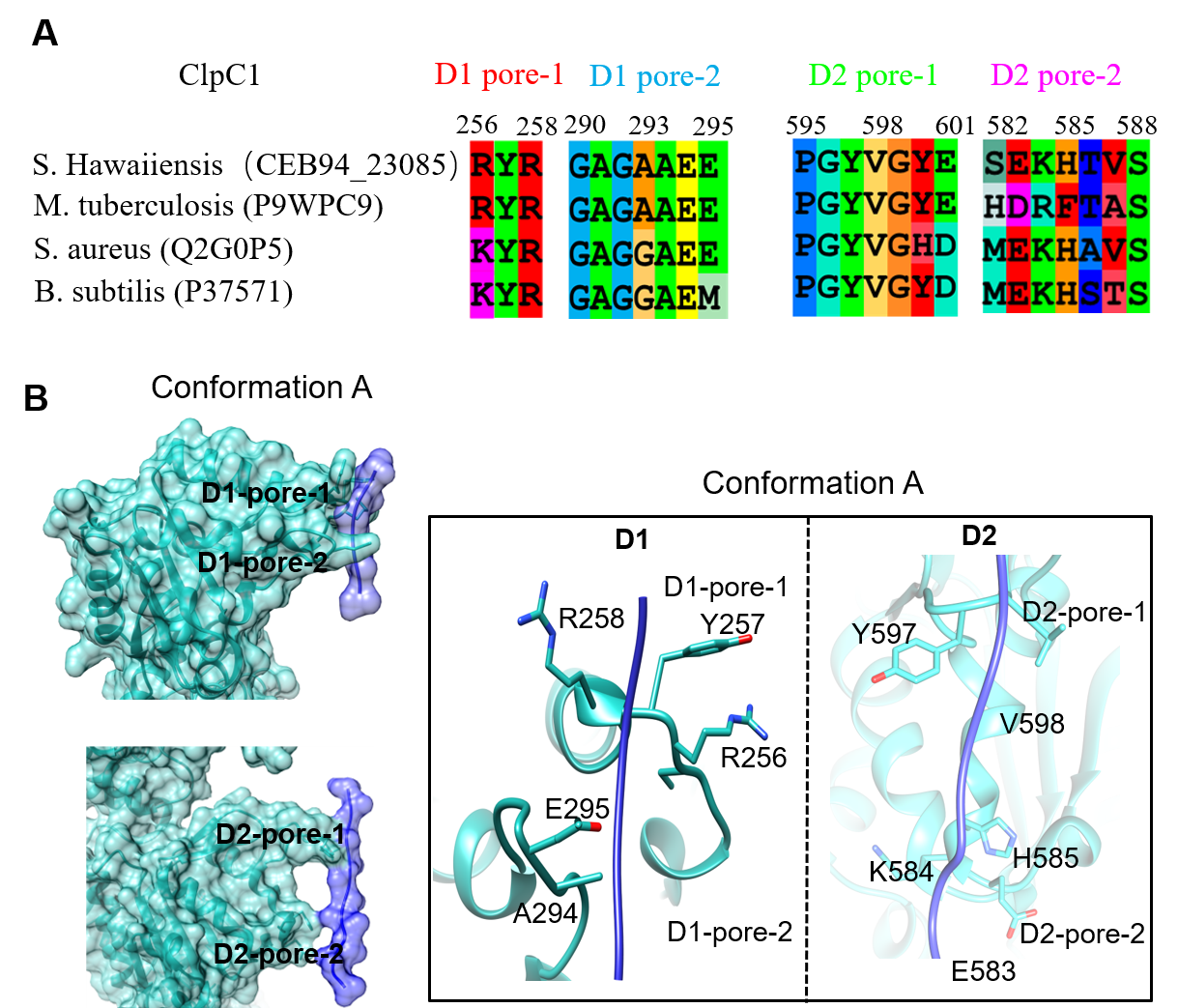


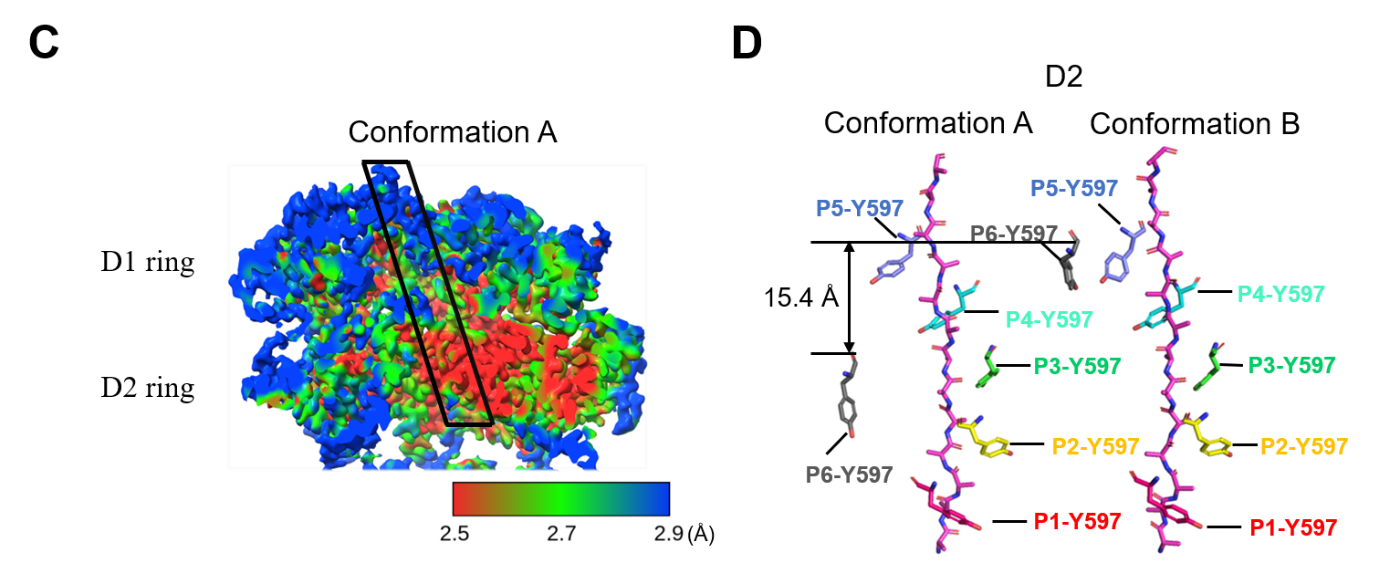


**Fig. S8 Substrate engagement by the D1 and D2 pore loops of ClpC1. A,** Multiple sequence alignment of the ClpC1 homologues reveals the conservation of the D1 and D2 pore loops of ClpC1, which mainly include the D1 Tyr-containing pore-1 loop (residues 256-258), D1 pore-2 loop (residues 290-295), D2 Tyr-containing pore-1 loop (residues 595–601), and D2 pore-2 loop (residues 582–588). **B,** Different interaction between the engaged substrate (bright blue) and the D1/D2 pore loops (cyan) of ClpC1. As an example, the vertical dissection of the substrate-bound ClpC1 in conformation A is shown. The major residues that aid in the substrate binding are labeled. The residues R256, Y257, R258 of the D1 pore-1 loop and the residues A294, E295 of the D1 pore-2 loop of ClpC1 help to stabilize the polypeptide substrate. The conserved residues Y597 and V598 of the canonical D2 pore-1 loop form a clamp around the substrate backbone, intercalating between side chains of the substrate. The residues E583, K584, and H585 of the D2 pore-2 loop project into the central channel and appear to directly interact with the substrate polypeptide. **C,** Local resolution estimation of the substrate-bound ClpC in conformation A indicates that the resolution of the D2 region is slightly higher than that of the D1 region. The position of the bound substrate is indicated by the black frame. **D,** Positional difference of the D2 pore-1 loop of the ClpC1 P6 protomer in conformation A and conformation B. The critical residue Y597 for substrate engagement is used to indicate the position of the D2 pore-1 loop along the substrate (magenta sticks).


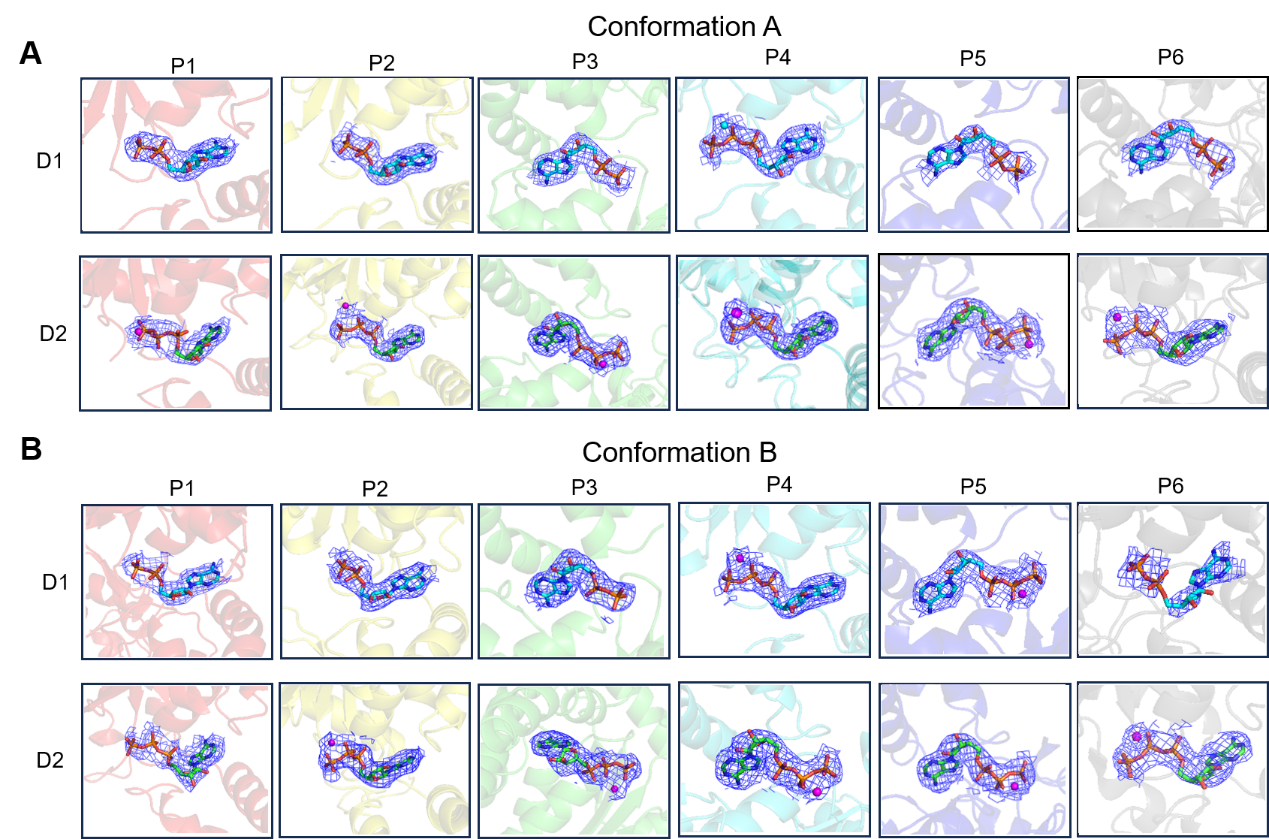


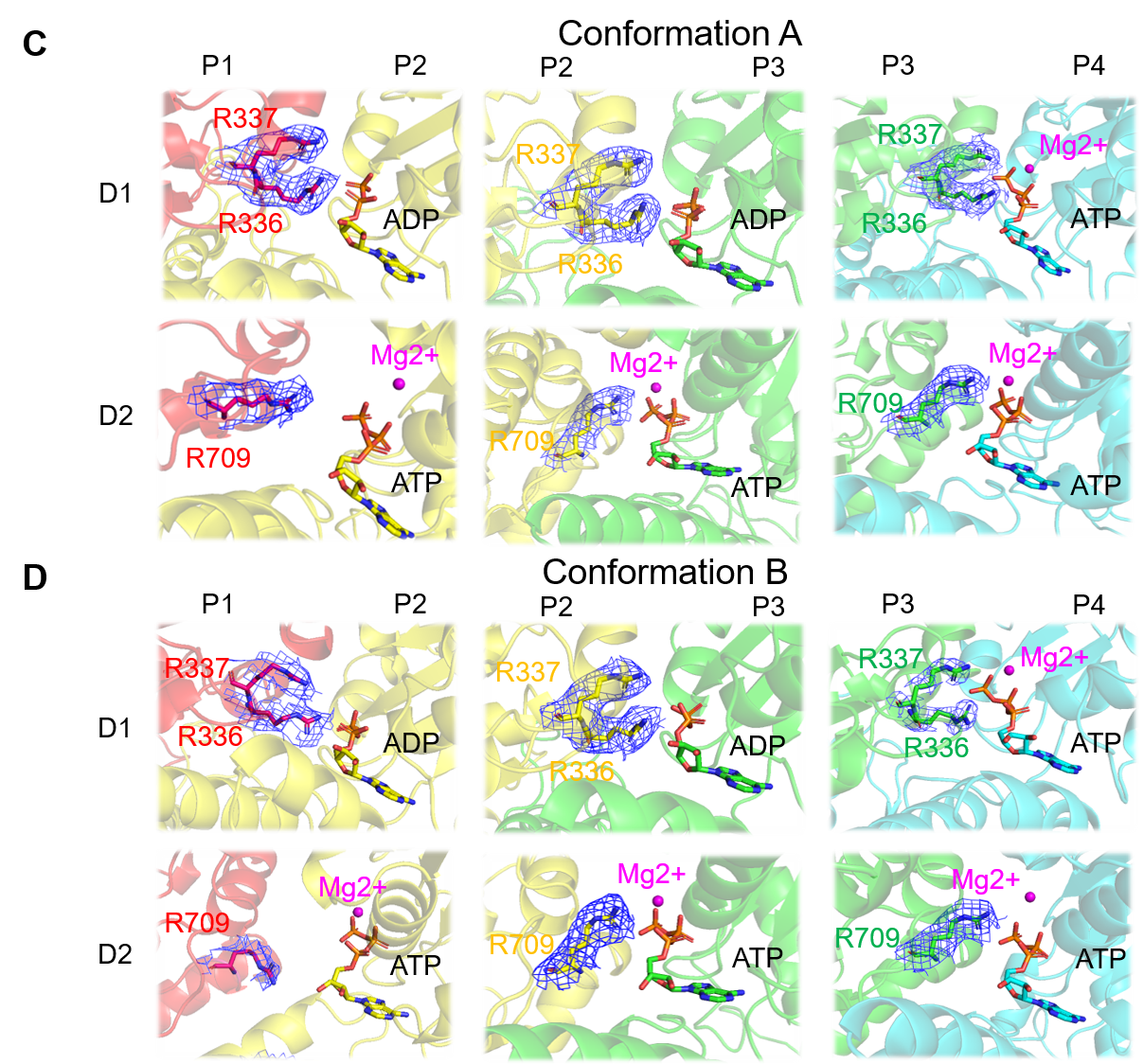


**Fig. S9 Nucleotide states of the ClpC1 protomers in two different conformations of the ClpC1:ClpP1P2 complex. A-B,** The bound ATP or ADP molecules are identified in the tandem ATPase domains (D1 and D2) of the ClpC1 protomers in conformation A **(A)** and conformation B **(B)**. The ligand density is contoured at 5σ. A magnesium ion (magenta ball) is always coupled to an activated ATP molecule bound in the catalytic center. **C**-**D**, Configuration comparison of the arginine fingers in the vicinity of the bound nucleotides in the ClpC1protomers of conformation A **(C)** and conformation B **(D)**. The arginine fingers of ClpC1 are identified as R336-R337 (D1 domain) and R709 (D2 domain) residing near the ATP-binding pockets at the interfaces between two adjacent ClpC1 protomers. The D1 and D2 arginine fingers of ClpC1 are shown in the cryo-EM density contoured at 3σ, respectively.

**Supplementary Video 1 legend:**

**Supplementary Video 1. Structural transition of the ClpC1:shClpP1P2 machinery.**
